# Misdiagnosis in occupational and environmental medicine: a scoping review

**DOI:** 10.1186/s12995-021-00325-z

**Published:** 2021-08-24

**Authors:** Jinyoung Moon, HyeKyoung Yoo

**Affiliations:** 1 Department of Environmental Health Science, Graduate School of Public Health, Seoul National University, Gwanak-ro 1, Gwanak-gu, Seoul, 08826 South Korea; 2 Department of Occupational and Environmental Medicine, Seoul Saint Mary’s Hospital, Banpo-daero 222, Seocho-gu, Seoul, 06591 South Korea

**Keywords:** Occupational and environmental medicine, Occupational disease, Environmental disease, Misdiagnosis, Scoping review

## Abstract

**Introduction:**

There has been no comprehensive review for misdiagnosis in Occupational and Environmental Medicine (OEM). The possible ramifications of an occupational disease (OD) or an environmental disease (ED) misdiagnosis are not just confined to the individual case but may extend to others exposed to the occupational or environmental hazard. Therefore, a comprehensive scoping review of published literature is imperative for understanding the nature of misdiagnoses in OEM.

**Methods:**

A medical librarian searched MEDLINE (PubMed), EMBASE, and the Cochrane Library (on 06 November 2020). All collected OEM misdiagnoses were classified based on 2 conceptual frameworks, the typical framework, and the causation model. The distribution of misdiagnosis across each medical specialty, each diagnostic step of the typical framework and the causation model, and false-negative and false-positive were summarized.

**Results:**

A total of 79 articles were included in the scoping review. For clinical specialty, pulmonology (30 articles) and dermatology or allergy (13 articles) was most frequent and second-most frequent, respectively. For each disease, occupational and environmental interstitial lung diseases, misdiagnosed as sarcoidosis (8 articles), and other lung diseases (8 articles) were most frequent. For the typical framework, the most vulnerable step was the first step, evidence of a disease (38 articles). For the causation model, the first step, knowledge base, was the most vulnerable step (42 articles). For reported articles, the frequency of false-negative (55 articles) outnumbered the frequency of false-positive (15 articles).

**Discussion:**

In OEM, compared to general medicine, causal misdiagnosis associated with the probability of causation is also important. For making a diagnosis in OEM, a knowledge base about possible ODs and EDs is essential. Because of this reason, the education and training of treating physicians for common ODs and EDs are important. For ODs and EDs, various intentional behaviors of stakeholders should be considered. This scoping review might contribute to the improvement of understanding for misdiagnosis in OEM.

**Supplementary Information:**

The online version contains Supplementary theoretical review and Supplementary materials available at 10.1186/s12995-021-00325-z.

## Introduction

Misdiagnosis in medicine is encountered in everyday medical practice. Misdiagnosis is generally more common in clinical specialties (e.g., emergency medicine and internal medicine) than in perceptual specialties (e.g., radiology and pathology) [1]. In a study in Britain, about 6% of admitting diagnoses were incorrect in British hospitals [2]. The specialties requiring complex decision-making in settings of above-average uncertainty and stress (for example, emergency medicine) reported up to a 12% diagnostic error rate [3, 4]. Based on lifelong studies on diagnostic decision-making, Arthur Elstein concluded that the rate of diagnostic error might be about 10–15% in overall medical practice [5].

Even if based on epidemiologic and toxicological principles, as a clinical specialty, occupational and environmental medicine (OEM) practice also encounters numerous instances of misdiagnosis. Compounding the opportunity for error, OEM practitioners have the added diagnostic step of detecting causality in addition to making the current medical diagnosis. Furthermore, the possible ramifications of an occupational disease (OD) or an environmental disease (ED) misdiagnosis are not just confined to the individual case but may extend to others exposed to the occupational or environmental hazard. Economic compensation and malpractice suits related to an OD or ED are other contributing factors to this complexity. For these reasons, the potential burden of misdiagnosis of an OD or ED could go beyond purely medical consequences, compared to typical misdiagnoses in general medicine. Because of these characteristics, various intentional behaviors of multiple stakeholders can possibly obscure the establishment of reliable causation between an occupational or environmental exposure and a related OD or ED [6–8].

To date, there has been no comprehensive review for misdiagnosis in OEM. However, with the potential wider implications of OD and ED considered a comprehensive review for this topic is imperative. In this scoping review, the authors tried to organize stepwise frameworks for the diagnosis of an OD and ED. Utilizing these frameworks, the authors analyzed and classified collected articles. In addition, the distributions of misdiagnoses in OEM through each specialty of medicine and by false-negative and false-positive misdiagnoses were also addressed. By examining the overall distribution patterns of misdiagnoses in OEM, the readers of this scoping review can understand patterns of misdiagnoses in OEM and devise possible preventive measures for reducing them.

## Methods

### The definition of misdiagnosis in OEM

When the concept of misdiagnosis in OEM is considered, the definition must include 2 categories. The first (i) is misdiagnosis from the general medical perspective, or medical misdiagnosis: the degree to which the diagnostic criteria for other medical diseases are fulfilled. The second (ii) is the misdiagnosis from the causal inference perspective, or causal misdiagnosis: the degree to which the occupational or environmental hazardous exposures contributed to the development of a disease. For this class of misdiagnosis, the probability of causation should exceed 50% [9]. However, given the active criteria of published articles, the reporting of this second class of misdiagnosis in OEM is relatively scarce. Therefore, the main discussion of this class of misdiagnosis is reviewed in the discussion section.

### Research question of this scoping review

The research question of this scoping review was to examine the distribution of misdiagnosis in OEM using published literature. This distribution included medical specialty, each step of diagnosis in OEM, and false-negative/false-positive.

### Information sources and the selection of evidence sources

A literature search was conducted by a medical librarian (information specialist, N.K. commented in the Acknowledgement section) in the library of one author’s affiliation (Department of Occupational and Environmental Medicine, Seoul Saint Mary’s Hospital). The medical librarian searched MEDLINE (PubMed), EMBASE, and the Cochrane Library (on 06 November 2020). Additionally, the authors searched the 3 databases on 08 January 2021 to complement the search results. Detailed search terminologies and search queries are provided from Supplementary A-1 to A-3.

The inclusion and exclusion criteria were as follows: (i) The article deals with a misdiagnosis case or an issue related to misdiagnosis. (ii) The misdiagnosis dealt with is an OD or an ED. (iii) The misdiagnosis should have a meaning in the present time, considering changes in diagnostic criteria and technologies with time. (iv) Both false-negative and false-positive misdiagnoses were included. (v) The publication year should be from 1990 to the present time. (vi) If an author reported the same set of misdiagnosis series in a number of articles, only the most recent one was included. (vii) Literature in all languages was included.

### Data items

Study type, subject population, initial misdiagnosis, correct final diagnosis, whether the article deals with a false-negative or false-positive case, the specialty of the doctor who made the initial and final diagnosis were summarized. For the classification between false-negative and false-positive, the OD or ED became the standpoint for classification. The content of each article was summarized in a Supplementary theoretical review and Supplementary materials. Possible corrective strategies were also summarized in the table.

### Classification of collected misdiagnoses

All collected misdiagnoses were classified based on 2 conceptual frameworks for the classification of misdiagnoses in OEM, which were provided in subsection 2.1 and 2.2 of Supplementary theoretical review (Table 1).

**Table 1:** Typical framework and causation model for misdiagnoses of OD and ED

Typical framework						
	(i) Evidence of a disease	(ii) Evidence of hazardous exposures		(iii) Evidence of causal relationship		
Proper work	Looking for any evidence of a disease in a subpopulation (workplace or community)	Extensively searching for the evidence of possible hazardous exposures in the workplace or community in which patients developed		Based on so-far known medical and public health knowledge, calculate the probability of causation		
Flaw	Shortage in knowledge about possible occupational and environmental diseases Misidentification of a disease	Shortage in knowledge about hazardous occupational or environmental exposures Shortage of information about exact exposure status of a patient		Shortage in knowledge about the causal relationship between a hazardous exposure and a disease outcome		
Consequences	Missed diagnosis OD or ED diagnosed as another general medical disease	OD or ED diagnosed as another general medical disease Sometimes the diagnosis itself could be denied.		OD or ED diagnosed as another general medical disease The estimated probability of causation below 50%: no acknowledgment of an OD or ED		
**Causation model**						
	**(i) Knowledge base**	**(ii) Heuristics**	**(iii) Complete work-ups**	**(iv) Diagnosis**	**(v) Management**	**(vi) Feedback**
Proper work	The first case in a similar exposure group should be examined meticulously with the generation of the knowledge base.	The proper context should be generated based on recognized findings.	Complete work-ups should be done for the consideration of sufficient differential diagnoses.	A valid synthesis should be carried out.	Appropriate treatments and preventive measures should be given.	Appropriate feedback should be given to diagnosis and management.
Flaw	Shortage in knowledge Base	Faulty heuristics	Immature closure	Faulty synthesis	Bad management	No policy or social system for feedback
Consequences	The OD or ED diagnosis for a similar exposure group cannot be made.	An initial diagnosis will be incorrect	An important differential diagnosis cannot be included.	The final tentative diagnosis will be incorrect.	The treatment and preventive measures will fail	Proper feedbacks on diagnostic processes and a trial of other management options cannot be made.

### Data charting process

The distribution of misdiagnosis across each medical specialty, each diagnostic step of the typical framework and the causation model, and false-negative and false-positive were summarized in Table 3, Table 4, and Table 5, respectively.

## Results

### Selection of evidence sources

Detailed search processes are in Supplementary material A-4. By the medical librarian, a total of 1168 articles were searched. By the authors, a total of 799 articles were searched. After excluding duplication, the authors conducted a primary selection process using the title and abstract. After this process, only 262 articles, searched by the medical librarian, and 62 articles, searched by the authors, remained. A full-text review was conducted for these 262 and 62 articles. Finally, 76 articles remained. From the bibliographies of relevant articles, 3 articles were additionally searched. Finally, a total of 79 articles were included in this scoping review.

### The characteristics and summary of individual evidence source

The characteristics of all included articles are summarized in Supplementary material D. The study period spread from 1967 to 2018. For the specialty of the diagnosing doctor, the initial misdiagnosis category included OEM physicians only in 9 articles out of 79 articles, but the final correct diagnosis category included OEM physicians in 17 articles out of 79 articles. The summary of the final included articles is provided in Table 2. The most and second-most frequent type of study was case-report and case-series, respectively (27 and 25 articles). The third-most and fourth-most frequent type of study was narrative review and discussion paper, respectively (10 and 6 articles). The following study types were case-control study, cohort study, surveillance data analysis, survey, and exposure assessment in order of frequency (3, 3, 2, 2, and 1 article, respectively). The summary and possible corrective strategies for each article are provided in Supplementary material B.

**Table 2:** The summary of the final included articles

Study type	Number of studies	Clinical specialty (final correct diagnosis)	False (+) or (−)^a^	Typical framework^b^	Causation model^c^
Case-report	27	Pulmonology: 11 Dermatology: 1 Orthopedics or trauma: 10 Other specialties: 5	False (+): 7 False (−): 20	[1] Evidence of a disease: 14 [2] Evidence of hazardous exposures:12 [3] Evidence of causal relationship: 1	[1] Knowledge base: 15 [2] Heuristics: 6 [3] Complete work-ups: 4 [4] Diagnosis: 1 [6] Feedback: 1
Case-series	25	Pulmonology: 10 Dermatology: 2 Other specialties: 13	False (+): 5 False (−): 17 False (+) and (−): 3	[1] Evidence of a disease: 12 [2] Evidence of hazardous exposures: 8 [3] Evidence of causal relationship: 5	[1] Knowledge base: 12 [2] Heuristics: 4 [3] Complete work-ups: 6 [4] Diagnosis: 3
Case-control study	3	Pulmonology: 3	False (−): 3	[2] Evidence of hazardous exposures: 3	[1] Knowledge base: 3
Cohort study	3	Pulmonology: 2 Other specialties: 1	False (+): 1 False (−): 1 False (+) and (−): 1	[1] Evidence of a disease: 3	[1] Knowledge base: 1 [3] Complete work-ups: 1 [4] Diagnosis: 1
Surveillance data analysis	2	Pulmonology: 1 Other specialties: 1	False (−): 2	[1] Evidence of a disease: 1 [3] Evidence of causal relationship: 1	[1] Knowledge base: 1 [2] Heuristics: 1
Survey	2	Other specialties: 2	False (−): 1 False (+) and (−): 1	[1] Evidence of a disease: 1 [3] Evidence of causal relationship: 1	[2] Heuristics: 1 [6] Feedback: 1
Exposure assessment	1	Pulmonology: 1	False (−): 1	[2] Evidence of hazardous exposures: 1	[1] Knowledge base: 1
Discussion	6	Pulmonology: 3 Orthopedics or trauma: 1 Other specialties: 2	False (+): 1 False (−): 4 False (+) and (−): 1	[1] Evidence of a disease: 2 [2] Evidence of hazardous exposures:2 [3] Evidence of causal relationship: 2	[1] Knowledge base: 1 [2] Heuristics: 1 [3] Complete work-ups: 3 [4] Diagnosis: 1
Narrative review	10	Pulmonology: 5 Orthopedics or trauma: 1 Other specialties: 4	False (−): 7 False (+) and (−): 3	[1] Evidence of a disease: 5 [2] Evidence of hazardous exposures: 5	[1] Knowledge base: 8 [2] Heuristics: 1 [4] Diagnosis: 1

^a^False (+) or (−) for Occupational Disease or Environmental Disease Category.

^b^Typical framework in the Methods section 2.5. Typical framework for the diagnosis of OD and ED.

^c^Causation model in the Methods section 2.6. Causation model for misdiagnosis of OD or ED.

### Initial misdiagnosis, correct diagnosis, and the frequency for each clinical specialty

When classified according to each clinical specialty (Table 3), misdiagnoses were reported most frequently in pulmonology (30 articles), followed by dermatology or allergy (13 articles). Reports in poisoning (10 articles) and orthopedics or trauma (10 articles) were also common.

**Table 3:** Initial misdiagnosis, correct diagnosis, and the frequency for each clinical specialty

Clinical specialty (frequency)	Initial misdiagnosis	Correct diagnosis	Frequency
Dermatology or Allergy (13 articles)	Non-allergic irritant contact dermatitis	Allergic contact dermatitis (Occupational epoxy)	2
	Allergic contact dermatitis	Non-allergic contact dermatitis	3
	Laryngopharyngeal reflux	Allergic laryngitis (Occupational origin)	1
	Asthma	Irritant vocal cord dysfunction	2
	Reactive airway dysfunction syndrome		
	Occupational asthma	No asthmatic reaction	1
	Asthma or exercise-induced bronchoconstriction	No lung function abnormality	1
	Bronchial asthma (occupational origin)	Carcinoid syndrome	1
	Other respiratory diseases	Occupational asthma	2
	No asthmatic reaction		
Pulmonology (30 articles)	Sarcoidosis	Chronic beryllium disease	8
		Silicosis	
		Occupational interstitial lung disease	
	Asthma or asthmatic bronchitis	Hypersensitivity pneumonitis	2
	Idiopathic interstitial lung disease		
	Pneumoconiosis or other lung diseases	Pneumoconiosis or other lung diseases	2
	Idiopathic pulmonary fibrosis or other lung diseases (including Tuberculosis)	Occupational or environmental interstitial lung disease (pneumoconiosis, silicosis, silicotuberculosis, or pigeon fancier’s lung)	8
	Other lung diseases	Chronic terminal airways and parenchymal lung disease, including obliterative bronchiolitis (occupational origin)	2
	Other lung diseases		
	Typical lung cancer	Occupational lung cancer	1
	Asbestos-related malignant cancer	Bilateral parietal pleural plaque	3
	Benign asbestos pleurisy	Malignant mesothelioma	2
	Thoracic aortic rupture or dissection		
	Drug-resistant pneumonia	Allergic bronchopulmonary aspergillosis	1
	Chronic beryllium disease	Mycobacterium infection (Avium intracellulare)	1
Poisoning (10 articles)	Other diseases	Poisoning (Lead, Methyl iodide and manganese, N-hexane, and Mercury)	6
	Neurotoxicant exposure or primary psychiatric illness and multiple chemical sensitivity	Neurotoxicant exposure or primary psychiatric illness and multiple chemical sensitivity	1
	Neurotoxic disease	A naturally occurring nervous system disease, psychogenic illness (including cognitive malingering)	2
	Viral flu	Polymer fume fever	1
Orthopedics or trauma (10 articles)	Occupational overuse syndrome	Guyon’s canal syndrome	1
	Other bone abnormalities	Condensing osteitis of the clavicle	1
	Motor dysfunction disease	Musician’s focal dystonia	1
	Ulnar styloid fracture	Calcific tendinitis of the flexor carpi ulnaris	1
	Occupational injury and illness or Other diseases of non-occupational origin	Occupational injury and illness or Other diseases of non-occupational origin	1
	Carpal tunnel syndrome	Nonspecific activity-related arm pain	1
	Hand-arm vibration syndrome	Cold hemagglutinin disease	1
	Fractured orbital bone	A plastic foreign body (Occupational craniofacial injury)	2
	Retrobulbar hemorrhage/edema		
	A spiral fracture of the right tibia	Unrecognized foreign body	1
Infection (5 articles)	Toxoplasmosis	Whipple’s disease	1
	An insect bite or Child abuse	Phytophotodermatitis	1
	Other febrile diseases.	Tsutsugamushi disease (occupational origin)	1
	Varicella-zoster virus infection	No infection	1
	Virus infection	Leptospirosis	1
Others (5 articles)	Acute kidney injury	Normal kidney function	1
	Occupational noise-induced hearing loss or other hearing loss disease	Occupational noise-induced hearing loss or other hearing loss disease	1
	Other psychiatric or stress disorder	Workplace adjustment disorder	1
	Arterial gas embolism	Facial nerve baroparesis	1
	Pelvic infection	Corpus cavernosum thrombosis	1
General (6 articles)	Other diseases	Occupational diseases, general	3
	Environmental illnesses (including Minamata disease) or other diseases	Environmental illnesses or other diseases	2
	Asthma or allergic condition	Multiple chemical sensitivity	1

For each disease, the most frequently reported type was chronic beryllium disease, silicosis, and other occupational interstitial lung diseases (ILD) misdiagnosed as sarcoidosis initially (8 articles) and occupational or environmental interstitial lung disease misdiagnosed as idiopathic pulmonary fibrosis or other lung diseases (8 articles). Poisoning was also commonly misdiagnosed as another disease (6 articles).

### Classification according to each step of the typical framework and causation model

The searched articles were classified according to each step of the typical framework and causation model (Table 4). For the typical framework, the most vulnerable step was the first step, evidence of a disease (38 articles). The next vulnerable step was the second step, evidence of hazardous exposures (31 articles). The following vulnerable step was the third step, evidence of causal relationship (10 articles).

**Table 4:** Classification according to each step of two diagnostic models

Typical framework						
Each step of the typical framework	(i) Evidence of a disease		(ii) Evidence of hazardous exposures		(iii) Evidence of causal relationship	
Frequency	38		31		10	
**Causation model**						
**Each step of the causation model**	**(i) Knowledge base**	**(ii) Heuristics**	**(iii) Complete work-ups**	**(iv) Diagnosis**	**(v) Management**	**(vi) Feedback**
Frequency	42	14	14	7	0	2

For the causation model, the first step, knowledge base, was the most vulnerable step (42 articles). The next was the complete work-ups step (14 articles) and the heuristics step (14 articles). Diagnosis (7 articles) and feedback (2 articles) steps also reported misdiagnosis. The articles classified in each step are listed in Supplementary material C-1 and C-2.

### Classification according to false-negative and false-positive

The searched articles were classified according to false-negative and false-positive (Table 5). For reported articles, the frequency of false-negative (55 articles) outnumbered the frequency of false-positive (15 articles). Some articles reported misdiagnoses that could be false-negative or false-positive according to circumstances (9 articles). For example, chronic beryllium disease can be diagnosed as sarcoidosis, which is false-negative. In contrast, the pulmonary infection of *M. avium* intracellulare can be diagnosed as chronic beryllium disease, which is false-positive. As another example, occupational noise-induced hearing loss can be diagnosed as other sensorineural hearing losses, which is false-negative. In contrast, other sensorineural hearing losses can be diagnosed as occupational noise-induced hearing loss, which is false-positive.

**Table 5:** Misdiagnosis according to false-negative and false-positive

Clinical specialty (frequency)	False-negative (55 articles)	Both false-negative and -positive (9 articles)	False-positive (15 articles)
Pulmonology	Occupational asthma [10, 11] Chronic beryllium disease [12–16] Hypersensitivity pneumonitis [17–19] Pigeon fancier’s lung [20] Obliterative bronchiolitis [21] Malignant mesothelioma [22] Occupational lung cancer [23] Silicosis [24–27] Interstitial lung disease [28–32] Mesothelioma [33, 34] Allergic bronchopulmonary aspergillosis [35]	Pneumoconiosis [65] Interstitial lung disease [66, 67] Asbestosis [68]	Occupational asthma [74, 75] Bronchial asthma [76] Asbestos-related cancer [77] Chronic beryllium disease [78]
Dermatology	Allergic contact dermatitis [36, 37] Phytophotodermatitis [38] Allergic laryngitis [39]		Latex allergy reaction [79] Allergic contact dermatitis [80, 81]
Orthopedics or trauma	Musician’s focal dystonia [40] Calcific tendinitis of the flexor carpi ulnaris [41] A foreign body [42–44] Condensing osteitis in the clavicle [45]	Workplace injury and illness [69]	Hand-arm vibration syndrome [82] Occupational overuse syndrome [83] Carpal tunnel syndrome [84]
Other clinical specialties	Tsutsugamushi disease [46] Methyl iodide and Manganese poisoning [47] N-hexane poisoning [48] Lead poisoning [49, 50] Mercury poisoning [51, 52] Polymer fume fever [53] Irritant vocal cord dysfunction [54, 55] Multiple chemical sensitivity [56] Adjustment disorder [57] Whipple’s disease [58] Occupational disease, general [59–61] Facial nerve baroparesis [62] Leptospirosis [63] Corpus carvernosum thrombosis [64]	Noise-induced hearing loss [70] Minamata disease [71] Multiple chemical sensitivity [72] Environmental illness [73]	Acute kidney injury [85] Varicella-zoster virus infection [86] Neurotoxic disease [87] Cognitive malingering in toxic exposure [88]

The false-negative or false-positive was determined using an occupational or environmental disease as the standard.

## Discussion

In this scoping review, OEM misdiagnoses reported in published literature were summarized (a total of 79 articles). The major study type was case report and case series (27 and 25 articles, respectively). The initial diagnosis team included OEM physicians only in 9 articles, but the final diagnosis team included OEM physicians in 17 articles. For clinical specialty, pulmonology (30 articles) and dermatology or allergy (13 articles) specialty were most frequent. For each disease, occupational and environmental interstitial lung diseases (ILD), misdiagnosed as sarcoidosis (8 articles), and other lung diseases (8 articles) were most frequent. For the typical framework, the most vulnerable step was the first step, evidence of a disease (38 articles). For the causation model, the first step, knowledge base, was the most vulnerable step (42 articles). For reported articles, the frequency of false-negative (55 articles) outnumbered the frequency of false-positive (15 articles).

### ‘Medical misdiagnosis’ versus ‘causal misdiagnosis’: the probability of causation

As stated in subsection 2.1, misdiagnoses in OEM are classified into 2 classes: ‘medical misdiagnosis’ and ‘causal misdiagnosis.’ The published articles usually focused on the first ‘medical misdiagnosis’ cases, and ‘causal misdiagnosis’ cases were scarcely reported. The reason for this might be the difficulty in calculating a correct probability of causation [9]. The ‘medical misdiagnosis’ is rather clearly defined and can be identified easily. However, the ‘causal misdiagnosis’ is the main area in which various disputes about compensation occur [89]. Case by case and physician by physician, the calculated probability of causation could be different, and this differently calculated probability of causation causes a different decision whether or not that this disease is of an occupational or environmental origin. Detailed discussion is provided in subsection 3.1. of Supplementary theoretical review.

### Misdiagnosis in general medicine versus misdiagnosis in OEM

Compared to our analysis showing the most frequent step for misdiagnoses in OEM as the first ‘knowledge base’ step, misdiagnoses in general medicine were most frequent in the ‘synthesis of collected information’ step [90], which corresponds to the second, third, and fourth steps in our causation model. This difference is because OEM usually uses the type 2 systematic and analytic approach to make a diagnosis, while general medicine also uses the type 1 heuristic and intuitive approach more commonly than OEM [91]. This diagnostic feature of OEM makes the education and training of treating physicians (including general physicians) for the clinical manifestations and diagnostic clues of various occupational and environmental exposures essential [92]. Because of this diagnostic feature of OEM, the initial misdiagnosis category included OEM physicians as the diagnosing physician only in 9 articles out of 79 articles, but the final correct diagnosis category included OEM physicians as the diagnosing physician in 17 articles out of 79 articles in this study. Given that OEM physicians usually have more knowledge about the clinical manifestations and diagnostic clues of various ODs and EDs, this result can be understood.

### Intentional behaviors of stakeholders

An important feature of ODs and EDs is that there are various intentional behaviors of stakeholders [93]. Because practical benefits like worker’s compensation or accident and sickness benefits exist in most countries, cognitive malingering could be prevalent in a specific workplace or country. This also causes false-positive cases. On the other hand, for employers, concealing industrial accidents or occupational diseases is favorable for their profits. Therefore, they usually try to elucidate no relationship between a specific working environment and an OD. This is the same for most EDs [94]. For these ED cases, the citizen takes the role of workers, and the company or the government who is responsible for having made an environmental hazard acts in the role of employer.

In addition, some environmental illnesses do not have definitive diagnostic criteria, and this causes both false-negative and –positive misdiagnoses [71–73]. As the solution for Minamata disease [71], a quantitative score can be instituted for diagnosis. Discriminant values in principal component analysis or classic machine learning methods are good examples of calculating this score.

### Risk of bias: case report and case series studies

This scoping review included 25 case report studies and 25 case series studies, among 79 total included studies. With case reports and case series that have no comparison group, one cannot conclude the magnitude or the frequency of a particular type of misdiagnosis [95]. However, these study types could provide an overall picture of misdiagnosis profile in OEM. This scoping review could be a foundation for future quantitative studies about particular types of misdiagnoses in OEM.

### Other limitations of this study

Several limitations exist in this scoping review. First, this study only included published misdiagnosis cases. Therefore, there will be some degree of publication bias in reported misdiagnosis cases. In particular, misdiagnoses with significant consequences for the treating physician might not be reported in the literature. In addition, considering the aforementioned confusion between the probability of causation and relative risk, acknowledged OD or ED might represent only a small percentage of overall true OD or EDs. Of the 3 categories of misdiagnosis aforementioned, only the first and second categories would have been reported in the literature. The third ‘missed diagnosis’ category should receive greater scrutiny in future research.

Second, there would be numerous misdiagnosis cases that were not revealed because of dynamics in workplaces or intentional behaviors of employers or employees. Sometimes, the government of a country is unfavorable to OD or EDs for economic growth (particularly developing countries). In this culture, the diagnosis of an OD or ED cannot be properly made.

## Conclusion

In this scoping review, we surveyed the distribution of misdiagnosis articles through each medical specialty, false-negative or –positive, and each diagnostic step of OEM. In the discussion, several related concepts are discussed. In OEM, in contrast to general medicine, causal misdiagnosis associated with the probability of causation is also important. For making a diagnosis in OEM, a knowledge base about possible ODs and EDs is essential. Because of this reason, the education and training of treating physicians for common ODs and EDs are important. For ODs and EDs, various intentional behaviors of stakeholders should be considered. This scoping review might contribute to the improvement of understanding misdiagnosis in OEM.

## Supplementary Information

**Additional file 1.** Supplementary theoretical review

**Additional file 2.** Supplementary materials

## Data Availability

This article is a scoping review, and all data and materials can be acquired from the published articles searched in 3 databases (PUBMED, EMBASE, and the Cochrane Library).
