# Supplementary material for: Misdiagnosis in occupational and environmental medicine: a scoping review
Source: J Occup Med Toxicol. 2021 Aug 24;16:33. doi: 10.1186/s12995-021-00325-z (PMC8383455; doi:10.1186/s12995-021-00325-z)
Supplement: Supplementary file 1 — Additional file 1. Supplementary theoretical review [file 12995_2021_325_MOESM1_ESM.pdf]

SUPPLEMENTARY THEORETICAL REVIEW

1. A conceptual serial diagnostic framework in occupational and environmental medicine

The diagnostic processes in occupational and environmental medicine (OEM) are divided into 2 steps. The first step is to find the most plausible explanation for the observed findings from a patient with an occupational disease (OD) or environmental disease (ED). And then, the second step is the processes in which the various sub-components of the social system act as a mold for the diagnostic processes of an OD or ED.

1.1. To find the most plausible explanation for the observed findings

The diagnosis of an OD or ED involves identifying key features, applying comprehensive sound logics covering both explicit and implicit findings, synthesizing all these logics into a coherent story of an OD or ED development. These processes do not involve merely typical history taking and physical examination, but these processes also consider scantly observed findings from a patient importantly. These overall findings should be explained as a coherent story, based on the most appropriate disease development model and to the best of the knowledge of an OEM physician. Considering this characteristic, the diagnostic processes in OEM require complicated mental processes involving cognition and decision-making for what story could best explain the gathered findings from a patient.

For this complex mental process, ‘Thinking, Fast and Slow’ by a Nobel Laureate, Daniel Kahneman, could provide valuable insight into the process of how the actual diagnosis is made (1). In this book, two separate cognitive processes for diagnostic reasoning are proposed. The first process is the ‘Heuristic approach’ (intuitive type 1 approach), and the second process is the ‘Analytical approach’ (systematic type 2 approach) (Table 1).

Table 1. The characteristics of type 1 and type 2 cognitive process (1)

Cognitive style	Type 1, heuristic, intuitive	Type 2, systematic, analytical
Computational principle	Associative	Rule-based
Responsiveness	Passive	Active
Capacity	High	Limited
Cognitive awareness/control	Low	High
Automaticity	High	Low
Rate	Fast	Slow
Reliability	Low	High
Errors	Relatively common	Rare
Effort	Low	High
Emotional attachment	High	Low
Scientific rigor	Low	High

The type 1 heuristic approach involves associative reasoning, passive responsiveness, high automaticity, and fast response. However, the reliability is somewhat low, accompanying errors and biases. In contrast with the type 1 approach, the type 2 analytical approach involves rule-based reasoning, active responsiveness, low automaticity, and slow response. The reliability of this approach is high, accompanying small errors and biases.

Recent findings from brain science further support this two-diagnostic-pathway theory and propose a much deeper understanding of human cognitive processes. They indicate that cognition is not just a passive perception but rather a series of predictions and error-checking by the brain. These whole processes are for making the best explanatory model of the current situation. When no errors are encountered in these processes, the brain constructs these neuronal layers into a hierarchical model set. This structure stores the perceived information as a pattern. At a later time, when the brain encounters a similar situation, it uses the quick type1 heuristic processes to treat this situation.

1.2. The social system itself as a mold for the diagnosis of an OD or ED

For the presentation patterns of an OD or ED to be readily recognizable by the treating physicians, an adequate amount of knowledge for the OD or ED is a prerequisite. This knowledge includes (i)

the ideas on potential hazardous exposures, (ii) various deviations from the normal health status, and (iii) a causal relationship between the hazardous potential exposures and the deviation from the normal health status. Among these sets of knowledge, in particular, the second ‘various deviations from the normal health status’ is composed of 3 constituents: (i) what is the baseline normal health status, (ii) through what route do the persons were exposed to these hazardous exposures, (iii) if the persons had not been exposed to these hazardous exposures, how would the health status of exposed persons have changed.

To gather these types of knowledge, not only the processes for a conventional medical diagnosis but also other social context information (occupational and environmental conditions included) which might have contributed to the development or aggravation of an existing OD or ED is required. For gathering this information, the role of each sub-components of the social system is essential. These sub-components include healthcare agencies, research agencies, OD and ED compensation systems, adjudication systems, administration agencies, non-governmental organizations, all related policies, and political bodies. The activity of these sub-components and the network among these sub-components are essential for complete the overall diagnostic processes for an OD or ED. Figure 1 provides the role of these sub-components of the social system in the continuous diagnostic processes for an OD or ED.

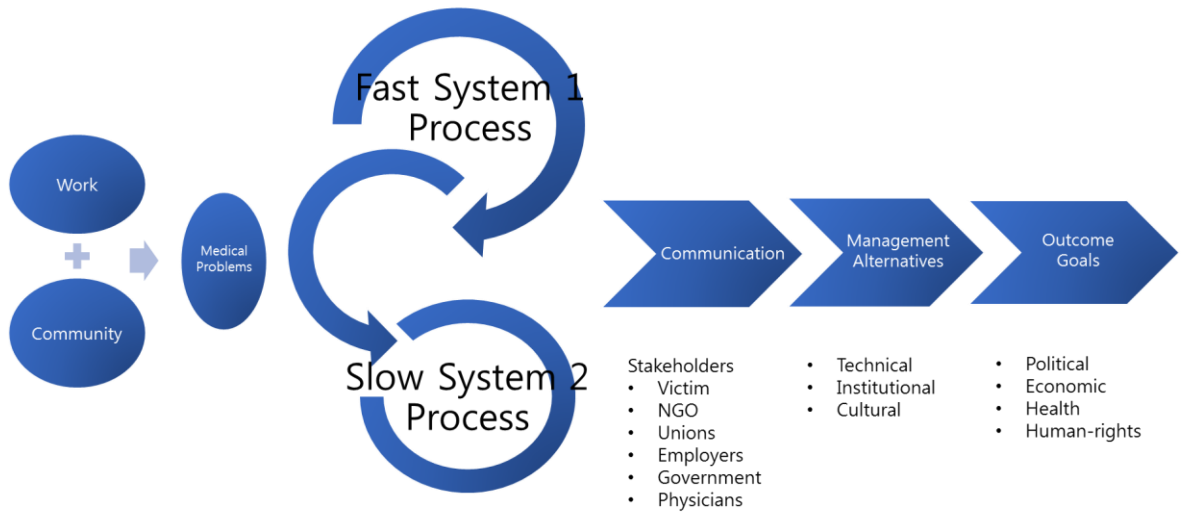

Figure 1. Diagnostic processes for an OD or ED: the role of the social system

Firstly, a suspected OD or ED patient from work or community gets into the healthcare system. In this system, the fast system 1 process and the slow system 2 process co-work to find out the possible explanation and the most plausible diagnosis for the patient. If a final OD or ED diagnosis is made, the knowledge about this diagnosis (epidemiology, risk factors, symptoms and signs, diagnosis, treatment, complications, and prognosis) is shared through various stakeholders. And then, technical, institutional, and cultural management alternatives are devised and applied to the population subjected to the same occupational or environmental exposures. Through these processes, the final outcome goals to achieve are political, economic, health, and human-rights improvements.

If available management alternatives or solutions do not exist, the identification of the OD or ED has no meaning in society. Therefore, usually, this OD or ED diagnosis is omitted. From this perspective, available technical, institutional, and cultural management options for an OD or ED themselves are encompassed in the diagnostic processes for the OD or ED. From this context, the pre-defined patterns between an OD or ED and the available management alternatives should be found in the society for the society to diagnose the OD or ED.

2. A classification framework for the causes of misdiagnoses in OEM

2.1. Typical framework for the diagnosis of OD and ED

The authors built up 2 conceptual frameworks for the classification of misdiagnoses in OEM based on previous literature (2-8). In the first 'typical framework', the diagnostic processes are divided into three serial steps (Table 2): (i) The first step is to look for any evidence of disease in a workplace or community. ('evidence of a disease') (ii) The second step is to extensively search for the evidence of possible hazardous exposures in the workplace or community in which a group of patients developed. ('evidence of hazardous exposures') (iii) The third step is to calculate the probability of causation based on so-far known medical and public health knowledge. ('evidence of causal relationship') According to each step of this typical framework, the selected articles were classified.

2.2. Causation model for misdiagnosis of OD or ED

Although a typical framework is devised for the cause of misdiagnosis in OEM to be explained, the framework is not sufficient. Therefore, the second framework 'causation model' for the classification of misdiagnoses in OEM according to the causes, was devised using the concepts and definitions introduced in articles that deal with misdiagnosis in general medicine (6-9) (Table 2).

In Table 2, the causation model is composed of six serial steps: (i) In the first 'knowledge base' step, if a physician has a deficient knowledge base for hazardous occupational and environmental exposures, the OD or ED diagnosis for a similar exposure group cannot be made. (ii) In the second 'heuristics' step, if faulty heuristics is applied, an initial diagnosis will be incorrect. (iii) In the third 'complete work-up' step, if complete work-ups are not conducted, a sufficient number of differential diagnoses cannot be included. (iv) In the fourth 'diagnosis' step, if faulty synthesis is applied, the final tentative diagnosis will be incorrect. (v) In the fifth 'management' step, if bad management is given, the treatment and preventive measures will fail. (vi) In the sixth 'feedback' step, if the culture

does not permit a diagnostic error or if there is a limited number of management alternatives, proper feedbacks to diagnostic processes and trial of other management options cannot be made. The selected articles were classified according to each step of this causation model.

Table 2. Typical framework and causation model for misdiagnoses in OEM

Typical framework			
	(i) Evidence of a disease	(ii) Evidence of hazardous exposures	(iii) Evidence of causal relationship
Proper work	Looking for any evidence of a disease in a subpopulation (workplace or community)	Extensively searching for the evidence of possible hazardous exposures in the workplace or community in which patients developed	Based on so-far known medical and public health knowledge, calculate the probability of causation
Flaw	Shortage in knowledge about possible occupational and environmental diseases<br>Misidentification of a disease	Shortage in knowledge about hazardous occupational or environmental exposures<br>Shortage of information about exact exposure status of a patient	Shortage in knowledge about the causal relationship between a hazardous exposure and a disease outcome
Consequences	Missed diagnosis<br>OD or ED diagnosed as another general medical disease	OD or ED diagnosed as another general medical disease<br>Sometimes the diagnosis itself could be denied.	OD or ED diagnosed as another general medical disease<br>The estimated probability of causation below 50%: no acknowledgment of an OD or ED

Causation model						
	(i) Knowledge base	(ii) Heuristics	(iii) Complete work-ups	(iv) Diagnosis	(v) Management	(vi) Feedback
Proper work	The first case in a similar exposure group should be examined meticulously with the generation of the knowledge base.	The proper context should be generated based on recognized findings.	Complete work-ups should be done for the consideration of sufficient differential diagnoses.	A valid synthesis should be carried out.	Appropriate treatments and preventive measures should be given.	Appropriate feedback should be given to diagnosis and management.
Flaw	Shortage in knowledge Base	Faulty heuristics	Immature closure	Faulty synthesis	Bad management	No policy or social system for feedback
Consequences	The OD or ED diagnosis for a similar exposure group cannot be made.	An initial diagnosis will be incorrect	An important differential diagnosis cannot be included.	The final tentative diagnosis will be incorrect.	The treatment and preventive measures will fail	Proper feedbacks on diagnostic processes and a trial of other management options cannot be made.

3. Several issues about misdiagnoses in OEM

3.1. ‘Medical misdiagnosis’ versus ‘Causal misdiagnosis’: the probability of causation

As stated in subsection 2.1, misdiagnoses in OEM are classified into 2 classes: ‘medical misdiagnosis’ and ‘causal misdiagnosis.’ The published articles usually focused on the first ‘medical misdiagnosis’ cases, and ‘causal misdiagnosis’ cases were scarcely reported. The reason for this might be the difficulty in calculating the correct probability of causation. The ‘medical misdiagnosis’ is rather clearly defined and can be identified easily. However, the ‘causal misdiagnosis’ is the main area in which various disputes about compensation occur (10). Case by case and physician by physician, the calculated probability of causation can be different, and this differently calculated probability of causation causes a different decision whether this disease is of an occupational or environmental origin or not.

For example, for radiogenic cancers in the US, the probability of causation/assigned share can be calculated using a web-based official tool (11, 12). In this case, the possibility of disputes for compensation is rare (mainly on the upper credibility limit of the probability of causation) (11). In South Korea, this criterion was applied successfully for occupational radiogenic cancers (13). For occupationally acquired contact dermatitis, the Mathias criteria can be applied to calculate the probability of causation (14). As seen in these examples, for some ODs or EDs with established criteria for the calculation of the probability of causation, the possibility of a subjective decision by a physician or a judge can be minimized. However, for most ODs and EDs, established criteria for the calculation of the probability of causation do not exist. Those cases are prone to disputes for the establishment of occupational or environmental causation.

3.2. The confusion between the probability of causation and rate fraction (attributable fraction)

The difference between the probability of causation and rate fraction (or attributable fraction) was dealt with in detail by Greenland (1999) (15). In the below equations, A_T is the total exposed

individuals, A_0 is the unaffected individuals, A_1 is the individuals with an accelerated disease occurrence, and A_2 represents the individuals with an all-or-none occurrence of a disease. These equations illustrate why these two concepts are different in algebraic terms.

$$\text{Rate Fraction (attributable fraction, attributable risk)} = A_2/A_T$$

$$\text{Probability of Causation (etiologic fraction)} = (A_1+A_2)/A_T$$

If the cases with accelerated occurrence are not considered, the rate fraction will be an underestimated value of the 'true' probability of causation. Even worse, for an accurate calculation of A_0, A_1, and A_2, a plausible biologic model is needed, based on enough domain knowledge on the biologic mechanisms of a disease occurrence.

For ODs and EDs, the standpoint for making a diagnosis should be the probability of causation and not the relative risk (or rate fraction). In other words, whether the probability of causation exceeds 50% or not should be the gold standard for making a specific OD or ED diagnosis. However, usually in legal actions, whether the exposure exceeded the doubling dose or not (relative risk over 2) is regarded as the typical standpoint for making a specific diagnosis of OD or ED. Relative risk over 2 (rate fraction of 50%) is not the same concept with the probability of causation over 50% because of the aforementioned unconsidered accelerated occurrence.

A more complicated problem is the variation of the probability of causation and rate fraction according to other associated factors like genetic variants or susceptibility factors. However, for the accurate estimation of the distribution of these two values, a complete understanding of the biological mechanisms of a disease occurrence is required.

3.3. Dose-response relationship and causal inference in OEM

For the calculation of a reliable probability of calculation, understanding the correct dose-response relationship between occupational or environmental exposure and an outcome is an essential

prerequisite. However, according to the exposure assessment method (16, 17) and applied biological dose-response model (18), the dose-response relationship can be markedly different. For example, a prospective cohort study revealed that cumulative vibration doses constructed from unweighted root-mean-square acceleration performed better for the prediction of vibration-induced white finger (19).

Inaccurate exposure assessment is another essential problem. Crude exposure assessment using only ever/never exposed or the duration of exposure cannot capture the accurate exposure dose of subjects. Particularly when dealing with subtle health effects, the intensity, duration, and route of exposure should be assessed meticulously to avoid an invalid dose-response relationship (20).

Finally, the interaction between a genetic factor (a susceptibility factor) and hazardous environmental exposure should be considered more thoroughly for causal inference. Some subpopulations with a genetic variant could be more susceptible to environmental or occupational exposure than other subpopulations (21). For these subpopulations, the dose-response pattern would be different from that for other subpopulations. In genetic epidemiology, this gene-environment interaction study has been conducted frequently (22). The relative risk distribution for environmental exposure would be varied according to each type of genetic variant, and this can be calculated and plotted using a recent Bayesian simulation technique (23). For an accurate dose-response relationship calculation and a correct causal inference, this genetic factor (susceptibility factor) and environmental exposure interaction should be considered in future studies.

3.4. Misdiagnosis in general medicine versus misdiagnosis in OEM

In our analysis, misdiagnosis in OEM was most frequent in the first step (knowledge base) of the causation model. The collection of appropriate exposure information and the OEM knowledge base of a treating physician was critical in making a correct diagnosis in OEM. However, according to Graber et al. (2005) (8), misdiagnoses in general medicine were more frequent in the 'synthesis of collected information' step, which corresponds to the second, third, and fourth steps in our causation model.

This difference is because OEM usually uses the type 2 systematic and analytic approach to make a diagnosis, while general medicine also uses the type 1 heuristic and intuitive approach more commonly than OEM (2). The heuristics approach makes the initial decision of physicians faster but is more susceptible to various cognitive pitfalls (6). Because OEM practice is based on epidemiologic principles, it requires complete information about the exposure status of a patient and a causal interpretation based on this information. This analytic trait of OEM makes OEM practices rely on the type 2 analytical approach more commonly than general medicine.

3.5. The role of education and training for treating physicians

Previous literature mainly focused on misdiagnoses in general medicine or internal medicine (3-6, 8). However, in this study, the authors focus on misdiagnoses in OEM. ODs and EDs usually have very different features compared to typical medical diseases: identifying potential hazardous occupational or environmental exposures is paramount in making a correct diagnosis. This includes an accurate exposure assessment for the patient. In addition, to confirm a causal relationship between exposure and a disease, the treating physician should have knowledge about possible occupational or environmental exposures. However, most physicians in the world are not receiving adequate education and training in OEM (24). Therefore, the whole process from the first to the third step in the typical framework does not work properly in reality, and an inaccurate diagnosis is made.

Because the typical framework is not sufficient to explain the observed misdiagnosis phenomenon in OEM, a causation model was devised by the authors. Although the Heuristics and Complete work-ups steps were responsible for some misdiagnosis cases, the fundamental problem of most misdiagnoses is the lack of knowledge base about OD and EDs. Without an adequate knowledge base about OEM, the treating physician cannot come up with potential differential diagnoses of ODs and EDs. This causes a significant cognitive trap in the diagnostic process. Faulty heuristics and a wrong decision not to conduct further diagnostic work-ups are usually caused by this insufficient knowledge base. The lack

of feedback on the tentative diagnosis or treatment and preventive measures is a problem of medical culture. Furthermore, with the unique and special characteristics of OD and EDs considered, this problem is also because of the lack of knowledge about OEM in treating physicians and policymakers.

Because of this pervasive lack of knowledge about OEM, false-negative misdiagnoses are common. Particularly without sufficient occupational or environmental history taking, a treating physician will miss the possibility of OD or ED and simply make a diagnosis of a general medical illness. However, there is also a possibility of false-positive diagnosis, particularly when a patient's occupational or environmental history is so distinctive that a treating physician falls into a heuristic trap. However, these cases were relatively uncommon than false-negative cases.

REFERENCES

1. Kahneman D, Egan P. Thinking, Fast and Slow (Farrar, Straus and Giroux, New York). Cited on. 2011:15.

2. Croskerry P. A universal model of diagnostic reasoning. Academic medicine. 2009;84(8):1022-8.

3. Willis CD, Mitton C, Gordon J, Best A. System tools for system change. BMJ Qual Saf. 2012;21(3):250-62.

4. Singh H, Graber ML, Kissam SM, Sorensen AV, Lenfestey NF, Tant EM, et al. System-related interventions to reduce diagnostic errors: a narrative review. BMJ Qual Saf. 2012;21(2):160-70.

5. Graber ML, Kissam S, Payne VL, Meyer AN, Sorensen A, Lenfestey N, et al. Cognitive interventions to reduce diagnostic error: a narrative review. BMJ Qual Saf. 2012;21(7):535-57.

6. Redelmeier DA. The cognitive psychology of missed diagnoses. Annals of internal medicine. 2005;142(2):115-20.

7. Rydon-Grange M. 'What's Psychology got to do with it?' Applying psychological theory to understanding failures in modern healthcare settings. J Med Ethics. 2015;41(11):880-4.

8. Graber ML, Franklin N, Gordon R. Diagnostic error in internal medicine. Archives of internal medicine. 2005;165(13):1493-9.

9. Kapur N. Mid Staffordshire hospital and the Francis Report: what does psychology have to offer. Psychologist. 2014;27:16-20.

10. Greenland S, Robins JM. Epidemiology, justice, and the probability of causation. Jurimetrics. 1999;40:321.

11. Kocher DC, Apostoaei AI, Henshaw RW, Hoffman FO, Schubauer-Berigan MK, Stancescu DO, et al. INTERACTIVE RADIOEPIDEMIOLOGICAL PROGRAM (IREP): A WEB-BASED TOOL FOR ESTIMATING PROBABILITY OF CAUSATION/ASSIGNED SHARE OF RADIOGENIC CANCERS. Health Physics. 2008;95(1):119-47.

12. Kocher DC, Apostoaei AI, Hoffman FO. RADIATION EFFECTIVENESS FACTORS FOR

USE IN CALCULATING PROBABILITY OF CAUSATION OF RADIOGENIC CANCERS. Health Physics. 2005;89(1):3-32.

13. Kim E-A, Lee E, Kang S-K, Jeong M. Probability of causation for occupational cancer after exposure to ionizing radiation. Annals of Occupational and Environmental Medicine. 2018;30(1).

14. Ingber A, Merims S. The validity of the Mathias criteria for establishing occupational causation and aggravation of contact dermatitis. Contact Dermatitis. 2004;51(1):9-12.

15. Greenland S. Relation of probability of causation to relative risk and doubling dose: a methodologic error that has become a social problem. American journal of public health. 1999;89(8):1166-9.

16. Jansen JP. Effects of measurement strategy and statistical analysis on dose-response relations between physical workload and low back pain. Occupational and Environmental Medicine. 2003;60(12):942-7.

17. Griffin MJ. Dose-response patterns for vibration-induced white finger. Occupational and Environmental Medicine. 2003;60(1):16-26.

18. Puskin JS. Perspective on the use of LNT for Radiation Protection and Risk Assessment by the U.S. Environmental Protection Agency. Dose-Response. 2009;7(4):dose-response.0.

19. Bovenzi M. A prospective cohort study of exposure-response relationship for vibration-induced white finger. Occupational and Environmental Medicine. 2010;67(1):38-46.

20. Semple S. Assessing occupational and environmental exposure. Occupational Medicine. 2005;55(6):419-24.

21. Demchuk E, Yucesoy B, Johnson VJ, Andrew M, Weston A, Germolec DR, et al. A Statistical Model for Assessing Genetic Susceptibility as a Risk Factor in Multifactorial Diseases: Lessons from Occupational Asthma. Environmental Health Perspectives. 2007;115(2):231-4.

22. Dennis J, Hawken S, Krewski D, Birkett N, Gheorghe M, Frei J, et al. Bias in the case-only design applied to studies of gene-environment and gene-gene interaction: a systematic review and meta-analysis. International journal of epidemiology. 2011;40(5):1329-41.

23. Mukherjee B, Ahn J, Gruber SB, Ghosh M, Chatterjee N. Case-Control Studies of Gene-

Environment Interaction: Bayesian Design and Analysis. Biometrics. 2010;66(3):934-48.

24. Rosenstock L, Rest KM, Benson Jr JA, Cannella JM, Cohen J, Cullen MR, et al. Occupational and environmental medicine: meeting the growing need for clinical services. New England Journal of Medicine. 1991;325(13):924-7.
