# Supplementary material for: Misdiagnosis in occupational and environmental medicine: a scoping review
Source: J Occup Med Toxicol. 2021 Aug 24;16:33. doi: 10.1186/s12995-021-00325-z (PMC8383455; doi:10.1186/s12995-021-00325-z)
Supplement: Supplementary file 2 — Additional file 2. Supplementary materials [file 12995_2021_325_MOESM2_ESM.pdf]

SUPPLEMENTARY MATERIALS

Supplementary material A-1. The search terminologies used by the medical librarian (N.K.) and the authors

Keywords	Search terminologies		
	MeSH	PubMed Entry Terms	Text Words
P) Occupational disease or Environmental disease or Occupational and environmental medicine	"Occupational Medicine"[Mesh]	Medicine, Occupational<br>Medicine, Industrial<br>Industrial Medicine	
	"Occupational Diseases"[Mesh]	Disease, Occupational<br>Occupational Disease<br>Occupational Illnesses<br>Illnesse, Occupational<br>Illnesses, Occupational<br>Occupational Illnesse<br>Diseases, Occupational	
	"Accidents, Occupational"[Mesh]	Occupational Accidents<br>Accident, Occupational<br>Occupational Accident<br>Accidents, Industrial<br>Industrial Accidents<br>Accident, Industrial<br>Industrial Accident	
	"Occupational Injuries"[Mesh]	Injuries, Occupational<br>Injury, Occupational<br>Occupational Injury	
	"Environmental Medicine"[Mesh]	Medicine, Environmental	Environmental disease
	"Environmental Health"[Mesh]	Environmental Healths<br>Healths, Environmental<br>Environmental Health Science<br>Environmental Health Sciences<br>Health Science, Environmental<br>Health Sciences, Environmental<br>Science, Environmental Health<br>Sciences, Environmental Health<br>Health, Environmental	
	“Occupational Health”[Mesh]		
	“Occupational Exposure”[Mesh]		
I) no keywords			
C) no keywords			
O) misdiagnosis	"Diagnostic Errors"[Mesh]	Errors, Diagnostic<br>Diagnostic Error<br>Error, Diagnostic<br>Misdiagnosis<br>Misdiagnoses	wrong diagnosis

Supplementary material A-2. The search results by the medical librarian (N.K.) on 06 November 2020

Database	Search	Search terminology / Search query	The number of searched articles
PubMed	#1	"Occupational Medicine"[Mesh]	23,345
	#2	"Occupational Medicine"[TW] OR "Medicine, Occupational"[TW] OR "Medicine, Industrial"[TW] OR "Industrial Medicine"[TW]	26,012
	#3	"Occupational Diseases"[Mesh]	132,725
	#4	"Occupational Diseases"[TW] OR "Disease, Occupational"[TW] OR "Occupational Disease"[TW] OR "Occupational Illnesses"[TW] OR "Illnesse, Occupational"[TW] OR "Illnesses, Occupational"[TW] OR "Occupational Illnesse"[TW] OR "Diseases, Occupational"[TW] OR "Occupational Disease"[TW]	85,848
	#5	"Accidents, Occupational"[Mesh]	17,456
	#6	"Accidents, Occupational"[TW] OR "Occupational Accidents"[TW] OR "Accident, Occupational"[TW] OR "Occupational Accident"[TW] OR "Accidents, Industrial"[TW] OR "Industrial Accidents"[TW] OR "Accident, Industrial"[TW] OR "Industrial Accident"[TW]	18,439
	#7	"Occupational Injuries"[Mesh]	2,905
	#8	"Occupational Injuries"[TW] OR "Injuries, Occupational"[TW] OR "Injury, Occupational"[TW] OR "Occupational Injury"[TW]	5,047
	#9	"Environmental Medicine"[Mesh]	459
	#10	"Environmental Medicine"[TW] OR "Medicine, Environmental"[TW] OR "Environmental disease"[TW] OR "Environmental diseases"[TW]	1,585
	#11	"Environmental Health"[Mesh]	25,398
	#12	"Environmental Health"[TW] OR "Environmental Healths"[TW] OR "Healths, Environmental"[TW] OR "Environmental Health Science"[TW] OR "Environmental Health Sciences"[TW] OR "Health Science, Environmental"[TW] OR "Health Sciences, Environmental"[TW] OR "Science, Environmental Health"[TW] OR "Sciences, Environmental Health"[TW] OR "Health, Environmental"[TW]	21,256
	#13 Combine	((((((((((("Occupational Medicine"[Mesh]) OR ("Occupational Medicine"[TW] OR "Medicine, Occupational"[TW] OR "Medicine, Industrial"[TW] OR "Industrial Medicine"[TW])) OR ("Occupational Diseases"[Mesh])) OR ("Occupational Diseases"[TW] OR "Disease, Occupational"[TW] OR "Occupational Disease"[TW] OR "Occupational Illnesses"[TW] OR "Illnesse, Occupational"[TW] OR "Illnesses, Occupational"[TW] OR "Occupational Illnesse"[TW] OR "Diseases, Occupational"[TW] OR "Occupational Disease"[TW])) OR ("Accidents, Occupational"[Mesh])) OR ("Accidents, Occupational"[TW] OR "Occupational Accidents"[TW] OR "Accident, Occupational"[TW] OR "Occupational Accident"[TW] OR "Accidents, Industrial"[TW] OR "Industrial Accidents"[TW] OR "Accident, Industrial"[TW] OR "Industrial Accident"[TW])) OR ("Occupational Injuries"[Mesh])) OR ("Occupational Injuries"[TW] OR "Injuries, Occupational"[TW] OR "Injury, Occupational"[TW] OR "Occupational Injury"[TW])) OR ("Environmental Medicine"[Mesh])) OR ("Environmental Medicine"[TW] OR "Medicine, Environmental"[TW] OR "Environmental disease"[TW] OR "Environmental diseases"[TW])) OR ("Environmental Health"[Mesh])) OR ("Environmental Health"[TW] OR "Environmental Healths"[TW] OR "Healths, Environmental"[TW] OR "Environmental Health Science"[TW] OR "Environmental Health Sciences"[TW] OR "Health Science, Environmental"[TW] OR "Health Sciences, Environmental"[TW] OR "Science, Environmental Health"[TW] OR "Sciences, Environmental Health"[TW] OR "Health, Environmental"[TW])	204,433
	#14	"Diagnostic Errors"[Mesh]	117,313
	#15	"Diagnostic Errors"[TW] OR "Errors, Diagnostic"[TW] OR "Diagnostic Error"[TW] OR "Error, Diagnostic"[TW] OR "Misdiagnosis"[TW] OR "Misdiagnoses"[TW] OR "wrong diagnosis"[TW]	53,413

	#16 Combine	("Diagnostic Errors"[Mesh]) OR ("Diagnostic Errors"[TW] OR "Errors, Diagnostic"[TW] OR "Diagnostic Error"[TW] OR "Error, Diagnostic"[TW] OR "Misdiagnosis"[TW] OR "Misdiagnoses"[TW] OR "wrong diagnosis"[TW])	132,509
	#17 Combine	#13 AND #16	547
	#18 Limit	#17 NOT ("animals"[MeSH] NOT "Humans"[MeSH])	545
Database	Search	Search terminology / Search query	The number of searched articles
EMBASE	#1	"occupational medicine"/exp	68,412
	#2	"Occupational Medicine":ti,ab,kw,de OR "Medicine, Occupational":ti,ab,kw,de OR "Medicine, Industrial":ti,ab,kw,de OR "Industrial Medicine":ti,ab,kw,de	28,634
	#3	"occupational disease"/exp	160,856
	#4	"Occupational Diseases":ti,ab,kw,de OR "Disease, Occupational":ti,ab,kw,de OR "Occupational Disease":ti,ab,kw,de OR "Occupational Illnesses":ti,ab,kw,de OR "Illnesse, Occupational":ti,ab,kw,de OR "Illnesses, Occupational":ti,ab,kw,de OR "Occupational Illnesse":ti,ab,kw,de OR "Diseases, Occupational":ti,ab,kw,de OR "Occupational Disease":ti,ab,kw,de OR "occupational disorder":ti,ab,kw,de OR "occupational dysfunction":ti,ab,kw,de OR "professional disease":ti,ab,kw,de	66,327
	#5	"occupational accident"/exp	27,040
	#6	"Accidents, Occupational":ti,ab,kw,de OR "Occupational Accidents":ti,ab,kw,de OR "Accident, Occupational":ti,ab,kw,de OR "Occupational Accident":ti,ab,kw,de OR "Accidents, Industrial":ti,ab,kw,de OR "Industrial Accidents":ti,ab,kw,de OR "Accident, Industrial":ti,ab,kw,de OR "Industrial Accident":ti,ab,kw,de OR "job-related accident":ti,ab,kw,de OR "job-related injury":ti,ab,kw,de OR "job-related trauma":ti,ab,kw,de OR "occupation related injuries":ti,ab,kw,de OR "occupation related injury":ti,ab,kw,de OR "occupational related injuries":ti,ab,kw,de OR "occupational related injury":ti,ab,kw,de OR "occupational trauma":ti,ab,kw,de OR "work-related accident":ti,ab,kw,de OR "work-related injury":ti,ab,kw,de OR "work-related trauma":ti,ab,kw,de	24,206
		"Occupational Injuries"	
	#7	"Occupational Injuries":ti,ab,kw,de OR "Injuries, Occupational":ti,ab,kw,de OR "Injury, Occupational":ti,ab,kw,de OR "Occupational Injury":ti,ab,kw,de	3,446
	#8	"environmental medicine"/exp	107
	#9	"Environmental Medicine":ti,ab,kw,de OR "Medicine, Environmental":ti,ab,kw,de OR "Environmental disease":ti,ab,kw,de OR "Environmental diseases":ti,ab,kw,de	4,756
	#10	"environmental health'/exp	40,106
	#11	"Environmental Health":ti,ab,kw,de OR "Environmental Healths":ti,ab,kw,de OR "Healths, Environmental":ti,ab,kw,de OR "Environmental Health Science":ti,ab,kw,de OR "Environmental Health Sciences":ti,ab,kw,de OR "Health Science, Environmental":ti,ab,kw,de OR "Health Sciences, Environmental":ti,ab,kw,de OR "Science, Environmental Health":ti,ab,kw,de OR "Sciences, Environmental Health":ti,ab,kw,de OR "Health, Environmental":ti,ab,kw,de	46,455
	#12 Combine	#1 OR #2 OR #3 OR #4 OR #5 OR #6 OR #7 OR #8 OR #9 OR #10 OR #11	277,759
	#13	"diagnostic error"/exp	102,339
	#14	"Diagnostic Errors":ti,ab,kw,de OR "Errors, Diagnostic":ti,ab,kw,de OR "Diagnostic Error":ti,ab,kw,de OR "Error, Diagnostic":ti,ab,kw,de OR "Misdiagnosis":ti,ab,kw,de OR "Misdiagnoses":ti,ab,kw,de OR "wrong diagnosis":ti,ab,kw,de OR "diagnosis error":ti,ab,kw,de OR "failure to diagnose":ti,ab,kw,de OR "false diagnosis":ti,ab,kw,de	76,676
	#15 Combine	#13 OR #14	116,920
	#16 Combine	#12 AND #15	759
	#17 Limit	#16 NOT ("animal"/exp NOT "human"/exp)	758
Database	Search	Search terminology / Search query	The number of searched articles

Cochrane Library	#1	[mh "Occupational Medicine"]	68
	#2	"Occupational Medicine":ti,ab,kw OR "Medicine, Occupational":ti,ab,kw OR "Medicine, Industrial":ti,ab,kw OR "Industrial Medicine":ti,ab,kw	140
	#3	[mh "Occupational Diseases"]	1,582
	#4	"Occupational Diseases":ti,ab,kw OR "Disease, Occupational":ti,ab,kw OR "Occupational Disease":ti,ab,kw OR "Occupational Illnesses":ti,ab,kw OR "Illnesse, Occupational":ti,ab,kw OR "Illnesses, Occupational":ti,ab,kw OR "Occupational Illnesse":ti,ab,kw OR "Diseases, Occupational":ti,ab,kw OR "Occupational Disease":ti,ab,kw	1,451
	#5	[mh "Accidents, Occupational"]	74
	#6	"Accidents, Occupational":ti,ab,kw OR "Occupational Accidents":ti,ab,kw OR "Accident, Occupational":ti,ab,kw OR "Occupational Accident":ti,ab,kw OR "Accidents, Industrial":ti,ab,kw OR "Industrial Accidents":ti,ab,kw OR "Accident, Industrial":ti,ab,kw OR "Industrial Accident":ti,ab,kw	158
	#7	[mh "Occupational Injuries"]	46
	#8	"Occupational Injuries":ti,ab,kw OR "Injuries, Occupational":ti,ab,kw OR "Injury, Occupational":ti,ab,kw OR "Occupational Injury":ti,ab,kw	523
	#9	[mh "Environmental Medicine"]	0
	#10	"Environmental Medicine":ti,ab,kw OR "Medicine, Environmental":ti,ab,kw OR "Environmental disease":ti,ab,kw OR "Environmental diseases":ti,ab,kw	43
	#11	[mh "Environmental Health"]	135
	#12	"Environmental Health":ti,ab,kw OR "Environmental Healths":ti,ab,kw OR "Healths, Environmental":ti,ab,kw OR "Environmental Health Science":ti,ab,kw OR "Environmental Health Sciences":ti,ab,kw OR "Health Science, Environmental":ti,ab,kw OR "Health Sciences, Environmental":ti,ab,kw OR "Science, Environmental Health":ti,ab,kw OR "Sciences, Environmental Health":ti,ab,kw OR "Health, Environmental":ti,ab,kw	148
	#13 Combine	#1 OR #2 OR #3 OR #4 OR #5 OR #6 OR #7 OR #8 OR #9 OR #10 OR #11 OR #12	2,780
	#14	[mh "Diagnostic Errors"]	2,771
	#15	"Diagnostic Errors":ti,ab,kw OR "Errors, Diagnostic":ti,ab,kw OR "Diagnostic Error":ti,ab,kw OR "Error, Diagnostic":ti,ab,kw OR "Misdiagnosis":ti,ab,kw OR "Misdiagnoses":ti,ab,kw OR "wrong diagnosis":ti,ab,kw	769
	#16 Combine	#14 OR #15	3,258
	#17 Combine	#13 AND #16	10
	#18 Limit	#17 NOT ([mh "animals"] NOT [mh "Humans"])	10

Supplementary material A-3. The additional search results by the authors on 08 January 2021

Database	Search	Search terminology / Search query	The number of searched articles
PubMed	#1	"Occupational Health"[Mesh]	34,176
	#2	"Occupational Exposure"[Mesh]	64,560
	#3 Combine	#1 OR #2	94,146
	#4	"Diagnostic Errors"[Mesh]	117,886
	#5	"Diagnostic Errors"[TW] OR "Errors, Diagnostic"[TW] OR "Diagnostic Error"[TW] OR "Error, Diagnostic"[TW] OR "Misdiagnosis"[TW] OR "Misdiagnoses"[TW] OR "wrong diagnosis"[TW]	53,843
	#6 Combine	("Diagnostic Errors"[Mesh]) OR ("Diagnostic Errors"[TW] OR "Errors, Diagnostic"[TW] OR "Diagnostic Error"[TW] OR "Error, Diagnostic"[TW] OR "Misdiagnosis"[TW] OR "Misdiagnoses"[TW] OR "wrong diagnosis"[TW])	133,352
	#7 Combine	#3 AND #6	224
	#8 Limit	#7 NOT ("animals"[MeSH] NOT "Humans"[MeSH])	224
Database	Search	Search terminology / Search query	The number of searched articles
EMBASE	#1	"occupational health"/exp	240,820
	#2	"occupational exposure"/exp	82,930
	#3 Combine	#1 OR #2	240,820
	#4	"diagnostic error"/exp	103,813
	#5	"Diagnostic Errors":ti,ab,kw,de OR "Errors, Diagnostic":ti,ab,kw,de OR "Diagnostic Error":ti,ab,kw,de OR "Error, Diagnostic":ti,ab,kw,de OR "Misdiagnosis":ti,ab,kw,de OR "Misdiagnoses":ti,ab,kw,de OR "wrong diagnosis":ti,ab,kw,de OR "diagnosis error":ti,ab,kw,de OR "failure to diagnose":ti,ab,kw,de OR "false diagnosis":ti,ab,kw,de	77,557
	#6 Combine	#4 OR #5	118,620
	#7 Combine	#3 AND #6	574
	#17 Limit	#16 NOT ("animal"/exp NOT "human"/exp)	574
Database	Search	Search terminology / Search query	The number of searched articles
Cochrane Library	#1	[mh "Occupational Health"]	670
	#2	[mh "Occupational Exposure"]	561
	#3 Combine	#1 OR #2	1,184
	#4	[mh "Diagnostic Errors"]	2,781
	#5	"Diagnostic Errors":ti,ab,kw OR "Errors, Diagnostic":ti,ab,kw OR "Diagnostic Error":ti,ab,kw OR "Error, Diagnostic":ti,ab,kw OR "Misdiagnosis":ti,ab,kw OR "Misdiagnoses":ti,ab,kw OR "wrong diagnosis":ti,ab,kw	788
	#6 Combine	#4 OR #5	3,285
	#7 Combine	#3 AND #6	1
	#8 Limit	#17 NOT ([mh "animals"] NOT [mh "Humans"])	1

Supplementary material A-4. The flow-chart for systematically collecting relevant articles

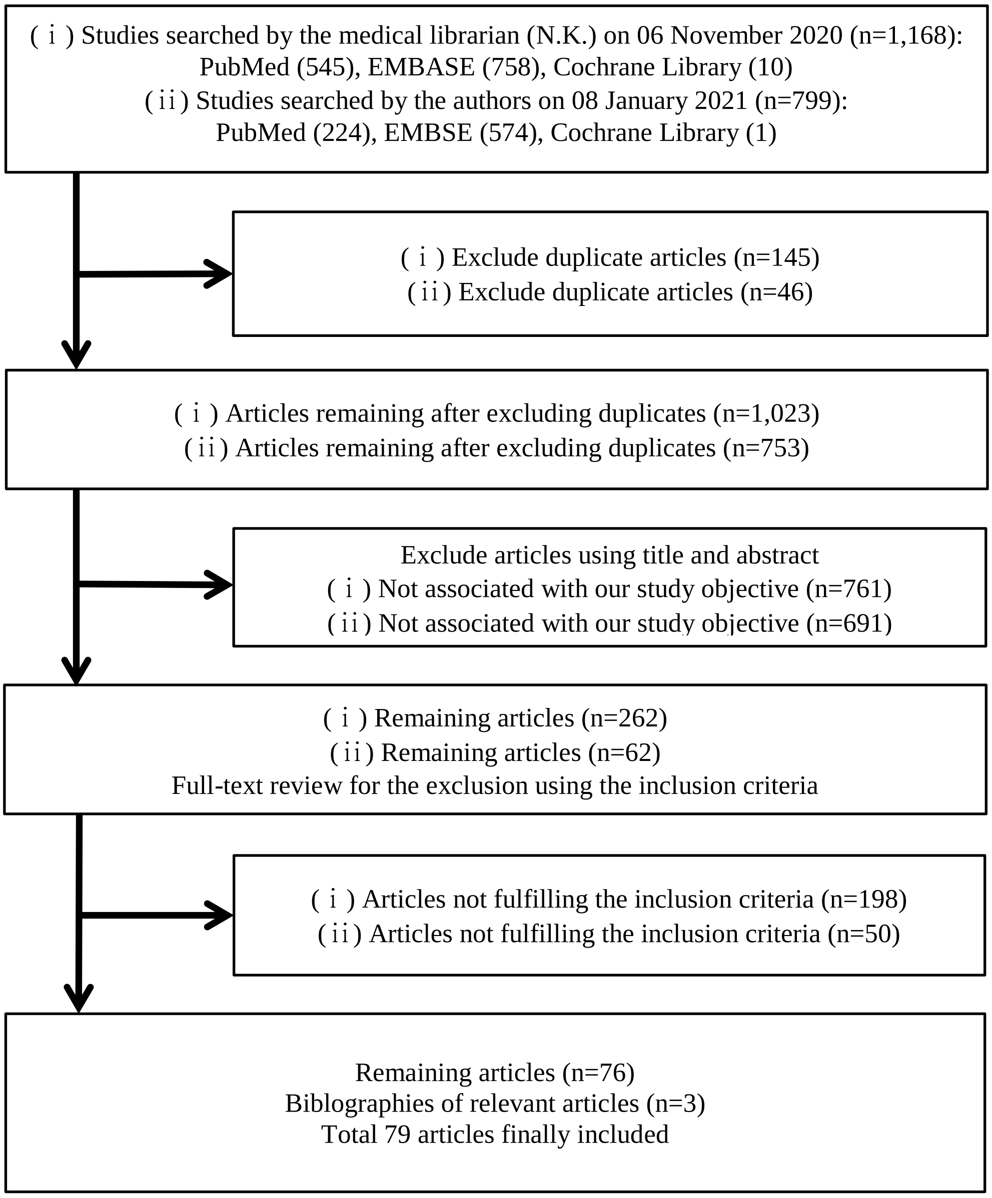

Supplementary material B. The summary of each article and possible corrective strategies

Article	Summary	Corrective strategies
Houle et al. (2012) (1)	"…25% of patients would have been misdiagnosed with nonallergic irritant contact dermatitis.", "…suffered from a misdiagnosis for a long period before being accurately diagnosed with allergic contact dermatitis." "…most of them not included in the commercially available allergens." By including actual workplace epoxy materials into patch tests, 13 patients attained additive value in diagnosing allergic irritant contact dermatitis. Particularly 6 of 13 patients gained a definitive additive value. (no reactions to commercially available allergens, but only positive reactions to custom epoxy materials)	The inclusion of actual workplace epoxy materials into the patch testing of patients with occupational epoxy exposure
Griffin et al. (2018) (2)	"Point of care (POC) testing under tropical field conditions routinely overestimates creatinine compared to laboratory testing, which leads to an overestimation of rates of acute kidney injury." "…POC consistently overestimated the creatinine by an average of 22% (95% CI: 19.8%, 24.7%), and the disagreement appeared greater at higher values of serum creatinine."	An adjustment factor of 0.7775 should be applied. This will lead to a significantly greater agreement between the two measures, point of care creatinine and serum creatinine. Rates of Acute kidney injury in the two combined groups fell from 72 to 57%.
Bruze et al. (2013) (3)	"On the basis of a positive cobalt spot test result, a woman was initially diagnosed with an occupational allergic contact dermatitis…" "No cobalt release was demonstrated with atomic absorption spectroscopy. (AAS)"	When a diagnosis of allergic contact dermatitis based on a positive spot test result is doubted, conformational analysis (like atomic absorption spectroscopy) should be performed.
Sastre et al. (2003) (4)	"In five patients who did not show an asthmatic reaction after the first specific inhalation challenge (SIC), PC20 exhibited more than a twofold reduction." "In three of the five patients, a second SIC with isocyanates elicited an immediate positive asthmatic reaction."	When a significant change in FEV1 is absent, PC20 should be systematically assessed before and after specific inhalation challenges with isocyanates to avoid false-negative results.

	Specific and nonspecific bronchial responsiveness may decline or disappear after cessation of exposure in the workplace in patients with occupational asthma, leading to false-negative specific inhalation challenge (SIC) results.	
Behrman et al. (2003) (5)	The original five latex bead agglutination assay (LA) results of 5 health care workers performed at hire were judged false-positives.<br>Of the 43 consecutive varicella-zoster viruses (VZV) LA samples, nine samples were false-positives.	LA is prone to false-positive and inappropriate for the screening of hospital healthcare workers.
Stenton et al. (1994) (6)	"The second series of double-blind inhalation challenges with carefully controlled exposures to glutaraldehyde (up to 0.32 ppm for 10min) gave rise to no obvious asthmatic reactions, in marked contrast to the results of the unblinded workplace challenge."<br>These results illustrate the potential for misdiagnosis of occupational asthma when unblinded challenge tests are used.	Inhalation challenge tests in a laboratory allow double-blinding and multiple-dose schedules. These can provide more objective evidence of occupational asthma.
Inai et al. (2009) (7)	"On the basis of various studies, pathological diagnosis in Japan at present includes misdiagnosis at the level of about 10％."	Efforts are necessary to improve the accuracy of the pathological diagnosis of mesothelioma using adequate immunohistochemical stains.
Ghio et al. (2014) (8)	"This approach employing optical microscopy is insensitive and leads to misdiagnosis."<br>Without the 'gold standard' histopathological examination of lung tissue, the diagnosis of interstitial lung disease (ILD) is being made. Even when tissue is available, the conventional light microscopic examination is only being used.	Scanning electron microscopy in combination with electron dispersive X-ray analysis should be employed whenever a possible relationship between interstitial lung disease (ILD) and particle or fiber exposure is doubted.
Muller et al. (2006) (9)	"In 84 patients seen for re-evaluation or making a diagnosis of sarcoidosis, beryllium exposure was recognized, and a diagnosis of chronic beryllium disease (CBD) was made in 34 out of 84 patients."	A detailed occupational history of sarcoidosis patients should be obtained to differentiate chronic beryllium disease from sarcoidosis.
Jacobs et al. (2005) (10)	"The acute presentation is almost always initially misdiagnosed as infectious in nature."<br>"…may respond partially to a bronchodilator, often leading to a misdiagnosis of asthma or asthmatic bronchitis."	Conduct natural or environmental challenges.<br>Consider that the clinical expressions of hypersensitivity pneumonitis could vary, affecting the entire respiratory system.

	"Criteria for diagnosis of hypersensitivity pneumonitis (HP) have been too restrictive and may have resulted in misdiagnosis."	The biopsy pattern for hypersensitivity pneumonitis can have all the histopathologic features of ILD.<br>Obtain environmental and occupational exposure histories of patients.
Garland et al. (2004) (11)	Military personnel who have worked aboard ships acquired a diagnosis of "sarcoidosis." However, the pathology review indicated a possible association between the history of assignments aboard ships and the presence of silica-like particles, titanium, and aluminum. (not simple sarcoidosis)	A detailed occupational history should be obtained.<br>Another diagnosis should be considered.
Fireman et al. (2003) (12)	Two of the 14 patients with evidence of granulomas in lung tissue (pulmonary involvement) and 1/14 with extrapulmonary involvement had a positive occupational exposure to beryllium and a positive beryllium lymphocyte transformation test (BeLTT) test. They had been erroneously diagnosed as having sarcoidosis instead of chronic beryllium disease.	Take a comprehensive occupational history in the clinical evaluation of patients suspected of sarcoidosis.
Kucenic et al. (2002) (13)	"Failure to test with the proper allergen(s) will lead to a misdiagnosis."<br>"In one study, 33 of 100 patients required testing with additional substances beyond the European standard series for the detection of their allergic occupational contact dermatitis. (OCD)"	Patch tests with a wide array of allergens than those currently available are needed to prevent the misdiagnosis of occupational contact dermatitis.<br>Physicians need to know the possible allergens and sources of exposure in workplaces.
Hartman et al. (1998) (14)	"…may be diagnosed on the basis of the subjective complaint, without taking into account toxic exposure history."<br>"They have easily missed diagnoses when toxic exposure mimics more commonly seen disorders."<br>"…cases of claimed toxic poisoning, with complaints made "legitimate" by repeated medical case note reference or a pseudo-diagnosis, i.e., multiple chemical sensitivity (MCS).<br>Exposure to a variety of neurotoxicants at work or in the home environment can elicit a	Diagnostic work-up, including a thorough psychiatric history, occupational, and environmental exposures, are required.<br>Physicians should be familiar with the subtle effects of common neurotoxicants.<br>Physicians are needed to have experience in patients with somatization and secondary gain intention.

	nonspecific symptom profile that is frequently mistaken for primary psychiatric illness. Alternatively, there are functional psychiatric states where patients resolutely insist on strict unifactorial organic causation but that are either functional or else deliberately manufactured for secondary gain.	
Kotloff et al. (1993) (15)	Chronic beryllium disease may be easily confused with sarcoidosis.	The knowledge about the relationship between beryllium exposure and chronic beryllium disease<br>Comprehensive history taking and the confirmatory test (beryllium lymphocyte transformation test) should be conducted for a suspected case.
Igata et al. (1993) (16)	"…but it is not always easy to diagnose in mild cases since the health of those affected ranges from severely damaged to healthy."<br>"Therefore, point D was settled on as the most reasonable dividing point to minimize misdiagnosis."<br>For environmental diseases like Minamata disease, misdiagnoses for mild cases could be common.	For the diagnosis of environmental disease, quantitative evidence is necessary so that everyone can agree with the diagnosis.<br>A meticulous investigation into various symptoms and signs of an environmental disease is needed.<br>For diagnosis, quantitative scores for a composite symptom or sign complex can be calculated. (for example, a discriminant value in principal component analysis) Recently, machine learning methods can also be applied.
Black et al. (1993) (17)	"Research has shown that individuals receiving a diagnosis of EI frequently have common psychiatric or medical disorders, which are usually recognized and untreated. Thus, the clinical ecologists are misinterpreting common signs and symptoms of illness and failing to prescribe appropriate and proven therapies."<br>The media are always interested in unusual stories that will attract attention and increase readership.<br>The public has been sensitized to the dangers of pollution and frightened by the news of environmental dangers.<br>EI patients become emotionally invested in the concept of their diagnosis and tend to be rejecting of alternate explanations for their symptoms, particularly psychological ones.	An acceptable case definition for an environmental illness is required.<br>The validity of a diagnostic method should be adequately demonstrated (e.g., reproducible laboratory test results, definitive physical abnormalities, characteristic symptom patterns, courses or outcomes, or response to treatment).<br>The pathologic mechanism of an environmental disease should be compatible with already known medical knowledge.<br>The provocation or neutralization testing method should be proven.

Kuratsune et al. (1974) (18)	In a town in Japan, a high proportion of males (ex-smelters in a local copper refinery) had been diagnosed with lung cancer. None of them had been considered as having occupational cancer by the physicians who treated them, their previous employer, the authorities concerned, their family and friends, or indeed themselves until this case-control study was conducted.	A lifelong health care system for ex-smelters from copper refineries is necessary.
Kerget et al. (2019) (19)	"Not taking appropriate occupational history by a physician may cause misdiagnosis or underdiagnosis of silicosis."<br>"Gender bias may be one of the reasons that her occupational history was not taken."<br>A female worker in a small-scale sandblasting factory was diagnosed with silicosis ten years later after her first symptoms.	Comprehensive history taking for silica exposure, compatible clinical, radiological, and pathological findings should be quickly obtained.<br>Physicians should be aware of the existence of unconscious gender bias.
He et al. (2019) (20)	The diagnosis of Tsutsugamushi disease was delayed because the clinician neglected the occupational history and lacked knowledge about the prevention and control of Tsutsugamushi disease.	Occupational history is crucial for the diagnosis of occupational-related diseases.
Wuellner et al. (2018) (21)	Many employers possess a limited understanding of workplace injury and illness recordkeeping requirements, potentially leading them to over-report minor incidents and under-report missed work cases.	The government should provide employers with education about recordkeeping methods for occupational injuries and illnesses.
Waljee et al. (2018) (22)	"…when the prevalence of a condition is low, and there are relatively few true positives while the number of false positives can be large…"<br>In low prevalence populations for idiopathic median nerve neuropathy at the carpal tunnel (carpal tunnel syndrome) like young workers, if the symptom is pain and no numbness, the diagnosis of nonspecific activity-related arm pain also should be considered.	Clinicians should understand the Bayes theorem.<br>Apply the Bayes theorem in calculating the possibility of true positive compared with false positive.<br>If a young worker is suspected of having carpal tunnel syndrome, periodically check out that the symptom is arm pain or numbness.<br>Do not refer to a surgeon immediately. For patients with mild symptoms, follow up with night splinting for years.
Dupas et al. (2013) (23)	Multiple chemical sensitivity (MCS) is often misdiagnosed as asthma or an allergic condition, which means that patients are frequently referred to as respiratory and allergy specialists. Misdiagnosis can lead to many futile medical investigations.	If the diagnosis of Multiple Chemical Sensitivity is suspected clinically, consider using the Environmental Exposure and Sensitivity Inventory (QEESI((c))) self-questionnaire.

Schaumburg et al. (2005) (24)	When an individual exposed to a putative neurotoxin presents associated symptoms, there are four possibilities: A genuine neurotoxic disorder, The coincident new onset of a naturally occurring nervous system disease, psychogenic illness, or worsening of a pre-existing neurologic or psychological disorder.	Patients with a suspected diagnosis of the neurotoxic disease could have another neurologic or psychological disease.
Egilman et al. (2004) (25)	After receiving a notification letter from the attorney of the occupational disease screening program, a 66-year-old man was preoccupied with the thought of an asbestos-related disease and committed suicide. However, the autopsy finding of the man revealed only bilateral parietal pleural plaques and no tumor or other abnormalities.	In-person medical counseling after screening programs<br>Prompt, accurate, and realistic counseling concerning the meaning of any positive findings (e.g., cancer)<br>Possible psychological consequences of any diagnosis should be considered by doctors.
Menezes et al. (2003) (26)	The lead poisoning case of an adult battery worker, who initially received symptomatic treatment because of clinical misdiagnosis<br>"For a long period of time, he remained asymptomatic. He was initially treated for his symptoms before the root cause of the problem was diagnosed."	Complete occupational and environmental exposure history is paramount.
Morgan et al. (1999) (27)	General practitioners (GP) usually do not have had any training in occupational medicine. Little contact between occupational health physicians and general practitioners also contributes to a lack of understanding of occupational diseases. These two factors could lead to a misdiagnosis of occupational diseases in GP practices.	Undergraduate and postgraduate training about occupational and environmental diseases<br>Professional contacts between occupational physicians and general practitioners should be encouraged.
Corbett et al. (1999) (28)	When compared with the autopsy findings, the radiological diagnoses of pneumoconiosis were not accurate. According to the change of diagnostic grades for pneumoconiosis, the sensitivity, specificity, positive predictive value, and negative predictive value changed.	Consider high-resolution computerized tomography (HRCT) or the typical computerized tomography (CT) scan for the diagnosis of pneumoconiosis cases.
Hinchcliffe et al. (1997) (29)	"The assessment of occupational noise-induced hearing loss is such a specialized subject that probably only customized training would be adequate."<br>The absence of education and training about occupational noise-induced hearing loss is an important factor causing medical examiner variability in accurately diagnosing occupational hearing loss.	Training and education on occupational noise-induced hearing loss<br>Occupational hearing loss should be included in education during undergraduate and postgraduate periods.<br>Appropriate consultation with occupational physicians
Laczniak et al. (2014) (30)	"Without a history of exposure to Beryllium, individuals with CBD have been	Beryllium exposure in occupational and environmental settings

	misdiagnosed as having sarcoidosis due to its very similar clinical presentation."<br>"Therefore, in order to reduce misdiagnoses and judge potential health risks, it is important first to determine if the potential exists for work-related or environmental Berilium exposure."	should be investigated meticulously for suspicious cases, particularly sarcoidosis.
Riario Sforza et al. (2017) (31)	"Hypersensitivity pneumonitis (HP) is often unrecognized and frequently misdiagnosed as respiratory infection or idiopathic interstitial lung disease."<br>The lack of widely accepted diagnostic criteria and diagnostic methods for HP<br>The lack of expertise for typical clinicians in hypersensitivity pneumonitis	Wider panels of the Immunoglobulin G test<br>Consultation with occupational physicians<br>Education and training for hypersensitivity pneumonitis
Kadu et al. (2012) (32)	"Many misdiagnoses and malpractices can occur due to unawareness of lead poisoning as an imitator of many organ symptoms."	A proper occupational history taking<br>Knowledge about the diagnosis and treatment of occupational lead poisoning
Glazer et al. (2011) (33)	"…Detailed mineralogical microanalysis of lung biopsies in patients diagnosed with IPF indicates that in 25% of the cases, occupational exposure was the likely cause."<br>"Accordingly, a missed inhalational exposure is the most frequent reason ILD specialty centers will disagree with the diagnosis from a referring community physician…"	A complete history taking about occupational and environmental exposures
Ansley et al. (2012) (34)	Sixty-five players with a physician diagnosis of asthma or exercise-induced bronchoconstriction (EIB) were referred for pulmonary function assessment. Among the tested, only 33 players (51%) had a positive bronchodilator or bronchial provocation test.	Objective pulmonary function test (PFT)
Dudarev et al. (2013) (35)	It is likely that the true burden of OD is higher than reported.<br>(i) The reluctance of workers concerned about job security to present for care, (ii) the lack of reliable information on working conditions needed to establish a causal link between disease and occupational exposure.	Accurate occupational history<br>A policy that guarantees the individual worker's job security<br>Fundamental reforms of the OD reporting system
Kahan et al. (1996) (36)	Family physicians and general practitioners both routinely asked patients about their job (78%) and exposure at work (75%), but at least in half the cases, this issue was not followed-up to determine if occupation-related counseling or problem management were necessary.	The attention of the society to occupational and environmental diseases

Constantin et al. (2015) (37)	Poor awareness of occupational exposure. (ignorance, under- or over-estimation)<br>Incorrect guiding of the occupational asthmatic patients to other health care services and not to the occupational medicine clinic<br>Communication impairments between the family doctor and enterprise doctor	A partnership collaboration and interdisciplinary teamwork between family doctors and occupational physicians
Guarnieri et al. (2019) (38)	Silicosis caused by inhalation of dust from artificial quartz conglomerates may exhibit atypical presentation and could be misdiagnosed as sarcoidosis (featuring occurrence in young workers, predominant localization in lung lymph nodes, and extrapulmonary involvement).	Accurate occupational history<br>General physicians should know this new hazardous occupational exposure.
Ross et al. (2016) (39)	Clinician's lack of familiarity with the potential toxicity of environmental and industrial chemicals can lead to misdiagnosis and mismanagement, and this lack of recognition can lead to continued exposure. (methyl iodide and manganese poisoning)	A detailed occupational history in patients who present with atypical neurological symptoms
Zhang et al. (2014) (40)	Sixteen cases of n-hexane poisoning were misdiagnosed as other diseases. 5 cases were misdiagnosed as Green Barry syndrome, 2 cases as motor neuron disease, 2 cases as drug-induced peripheral neuropathy, 3 cases as periodic paralysis, and 4 cases had an uncertain diagnosis.<br>Most doctors who work in ordinary hospitals do not know occupational chronic n-hexane poisoning.	Correct occupational history<br>Knowledge about the clinical features of n-hexane poisoning
Lu et al. (2013) (41)	A total of 1,178 cases of misdiagnosed pneumoconiosis or silicotuberculosis were collected. There were 13 causes of misdiagnosis, and the most common one was the poor ability of identification due to inadequate experience in reading chest X-ray films (45.93%), followed by the neglect of the patient's occupational history (44.99%). Other causes of misdiagnosis included complex X-ray findings that are difficult to judge (29.03%), poor quality of chest radiographs (23.09%), and lack of regular health supervision (19.95%).	Enhance the training and education about the diagnosis of pneumoconiosis
Sauler et al. (2012) (42)	The difficulties in recognizing the role of occupational and environmental exposures, including clinician awareness and recognition, misdiagnosis and limited information on	A high index of suspicion for the potential toxicity of occupational and environmental exposures

	work and environmental exposures, and the presentation of variable clinical phenotypes in response to a single exposure<br>(Chronic terminal airways and parenchymal lung disease)	
Baur et al. (2016) (43)	For a berylliosis case, based on the presence of bilateral hilar lymphadenopathy, the diagnosis of sarcoidosis was made at that time, without any histological investigations and without taking a detailed case history.	|A detailed history taking, including occupational history
Rodriguez et al. (2005) (44)	Musician's focal dystonia (MFD) is rarely diagnosed because those who experience it do not seek medical attention for fear or shame, but also because many physicians do not consider the disease in the differential diagnosis of motor dysfunction.	Musician's focal dystonia should be suspected when a musician reports an involuntary movement.
Shusterman et al. (1993) (45)	In polymer fume fever, constitutional signs and symptoms typically present several hours after initial exposure, often giving rise to a misdiagnosis of viral flu.	Careful history taking of occupational exposures
Ryan et al. (1993) (46)	The diagnosis of the fracture of the ulnar styloid was made. The patient was treated in a plaster cast and referred to the fracture clinic. When reviewed, the orthopedic surgeon made the correct diagnosis of calcific tendinitis of the flexor carpi ulnaris.	Knowledge about a specific occupational disease (calcific tendinitis of the flexor carpi ulnaris)
Tezer et al. (2011) (47)	"Since the mercury exposure was not known, the initial presentation and clinical picture suggested a misdiagnosis, a contagious infectious disease, because the onset of symptoms occurred at different times in the same family members."	Knowledge about the toxic properties of mercury and various clinical presentations of mercury poisoning
Tonini et al. (2009) (48)	In a subset of vocal cord dysfunction (VCD) patients, the disease can be attributed to occupational or environmental exposure to inhaled irritants.<br>A misdiagnosis with asthma is common. (by the paradoxical adduction of vocal cords during inspiration)	Strict compliance with the American Thoracic Society (ATS) criteria for spirometry<br>The morphologic analysis of flow-volume curves and flow-volume loop
Chirico et al. (2016) (49)	A significant challenging issue concerns the misdiagnosis of adjustment disorder because it is a longitudinal diagnosis based on etiology and outcome and because it has no reliable and valid diagnostic tool.<br>In Italy, occupational health surveillance programs are useless because the agreement between employers and persons delegated by workers may stop at the first step of the	Workplace health surveillance by an occupational physician can help the diagnosis of chronic adjustment disorder in workplaces.

	work-related stress risk assessment.	
Kales et al. (2006) (50)	"Cases 1 and 2 initially were misdiagnosed as suffering allergic reactions to natural rubber latex (NRL), resulting in 7- and 23-months lost time, $15,790 and $139,000 indemnity costs, respectively, and both failing to return to work."<br>The failure to recognize that synthetic paints do not contain NRL can lead to misdiagnosis, inappropriate exposure, and work avoidance recommendations, and iatrogenic disability.	The knowledge that synthetic paints do not contain natural rubber latex.<br>A thorough and prompt investigation of the actual exposures coupled with assertive education of the patient and their personal physician can reduce or avoid negative outcomes.<br>Advice and reassurance from physicians that encourage a return to work
Randhawa et al. (2010) (51)	"In our patient cohort, three times as many patients demonstrated allergy compared with laryngopharyngeal reflux. (LPR) This has led us to question if some patients with allergic laryngitis are being misdiagnosed with LPR."	The possibility of allergic laryngitis should be considered in laryngopharyngeal reflux patients.<br>Complete allergy studies should be conducted.
Ohtani et al. (2008) (52)	Two cases of chronic summer-type hypersensitivity pneumonitis were misdiagnosed as having idiopathic interstitial pneumonia.<br>The clinical features of chronic hypersensitivity pneumonitis (HP) are similar to idiopathic interstitial pneumonia. (IIPs)	Chronic summer-type hypersensitivity pneumonitis should be included in the list of differential diagnoses of chronic interstitial lung diseases.<br>Environmental investigation to find the antigen is important to make the patients avoid antigen exposures.
Chan et al. (2008) (53)	A case of Guyon's canal syndrome caused by a ganglion in a computer user was misdiagnosed as an occupational overuse syndrome. (OOS)	For a computer user with symptoms suspicious of occupational overuse syndrome, a nerve conduction or imaging study is necessary.
Taskar et al. (2006) (54)	Given the low utilization of the gold standard (a surgical lung biopsy) and the complexity of diagnosis, there is a high likelihood of misdiagnosis for IPF.	Electron microscopy and energy-dispersive X-ray analysis for the diagnosis of IPF patients<br>Environmental and occupational exposure history
Liu et al. (2011) (55)	Because of its diverse clinical symptoms, mercury poisoning was easy to misdiagnosis and missed diagnosis.	The knowledge about mercury poisoning
Galdi et al. (2005) (56)	A case of irritant vocal cord dysfunction at first misdiagnosed as reactive airway dysfunction syndrome. (RADS)	Irritant vocal cord dysfunction should always be considered for patients with acute respiratory symptoms after exposure to irritants

		and with asthma-like symptoms that fail to respond to conventional asthma therapy.
Mirzaei et al. (2019) (57)	In a 35-year-old male patient with an occupational craniofacial injury, a plastic foreign body (a piece of a plastic pipe) was removed from the orbital cavity, which was suspected to be a fractured orbital bone, in the first place.	Plastics sometimes mimic bone structures in a CT scan.<br>High clinical suspicion is necessary.
Papakonstantinou et al. (2017) (58)	Whipple's disease: a 22-year-old man with no risk factors was erroneously diagnosed with and treated for toxoplasmosis based on consistent lymph node histology. The correct diagnosis was delayed by the dramatic symptomatic improvement resulting from the previous therapy targeted to toxoplasmosis.	Occupational exposure history to soil or animals<br>Whipple's disease should be considered in cases of granulomatous lymphadenopathy of unknown cause, even if the age of the patient does not fit the classic presentation of the disease.
Mouawad et al. (2014) (59)	A woman visited with an acute onset of interscapular pain and hemodynamic instability. The radiographic imaging was suggestive of acute descending thoracic aortic rupture. However, the final diagnosis was advanced malignant mesothelioma.	Oncological thoracic pathology (e.g., malignant mesothelioma) should be considered in patients with signs and symptoms mimicking acute thoracic aortic rupture or dissection.
Poole et al. (2008) (60)	A patient with a diagnosis of hand-arm vibration syndrome was referred for a second opinion. He was finally diagnosed with idiopathic cold hemagglutinin disease.	Exposure to vibration can be confounded with exposure to cold.<br>Cold hemagglutinin disease or cryoglobulinemia should be excluded before making the diagnosis of hand-arm vibration syndrome.
Walusiak et al. (2002) (61)	A 25-year-old baker, who had worked in a bakery for ten years, had been mistakenly diagnosed with bronchial asthma rather than carcinoid syndrome. (Specific inhalation challenge with flour and bakers' additives did not induce any decrease in FEV1 and PEFR or increase in bronchial hyperreactivity)	Specific inhalation challenges should be the gold standard for the diagnosis of occupational asthma.
Greve et al. (2006) (62)	Based on the retrospective review of 128 neuropsychological cases with a financial incentive, the prevalence of malingering was 40% (based on clinical method) or from 30 to more than 45%. (based on statistical modeling)	Cognitive malingering should always be considered in toxic exposures.
Weber et al. (1999) (63)	Phytophotodermatitis is often misdiagnosed as an insect bite or child abuse in children. Particularly, the misdiagnosis of phytophotodermatitis in children could lead to inappropriate accusations of child abuse.	Complete exposure history and careful observation<br>In patients presenting with unusual appearing rash patterns, recent exposure to any citrus fruits should be asked.

Rosal-Sanchez et al. (2002) (64)	A 3-year-old child, who developed pigeon fancier's lung (PFL) after brief contact with pigeons, was misdiagnosed as tuberculosis initially.	Pigeon fancier's lung should be considered for patients with intermittent pulmonary and systemic symptoms of interstitial lung disease.<br>Correct laboratory analyses like specific IgG antibodies to pigeon serum, ELISA, lymphocyte transformation test, SDS-PAGE, and immunoblotting
Greenspan et al. (1991) (65)	Condensing osteitis of the clavicle can be mistaken for other bone abnormalities such as Friedrich disease, bone island, osteoid osteoma, sternoclavicular osteoarthritis, and even metastasis and osteosarcoma.	Condensing osteitis of the clavicle should be considered for sclerotic lesions at the sternal end of the clavicle, particularly in young and middle-aged women.
Mazzei et al. (2019) (66)	Occupational interstitial lung diseases (ILD): no standardized diagnostic criteria, varied physician awareness and training, limitations inherent to the various data sources, and the long latency period	Radiologists should recognize patterns of occupational ILD that can induce the emergence of hidden occupational histories.
Center for Disease Control and Prevention (2013) (67)	Two cases of obliterative bronchiolitis identified in workers employed in a small coffee-processing facility<br>Both patients' illness was misdiagnosed before they received a diagnosis of work related to obliterative bronchiolitis, which had not been identified previously in the coffee-processing industry.	Flavoring chemicals should be recognized as hazardous occupational exposure.<br>A high index of suspicion is required when potentially exposed workers show progressive shortness of breath.
Bouzgarrou et al. (2020) (68)	The diagnosis reviewed by the committee was an Irritant Contact Dermatitis for the majority of cases. The distinction between Allergic Contact Dermatitis and Irritant Contact Dermatitis is difficult for Occupational dermatitis cases.	Understanding a misdiagnosis between allergic contact dermatitis and irritant contact dermatitis can occur, even if the diagnosis was made based on exposure history, clinical examination, and skin prick tests.
Marinides et al. (2019) (69)	This case report describes recurrent facial baroparesis in a military diver, which manifested on the contralateral sides of his face. His initial presentation was misdiagnosed as an arterial gas embolism. Upon recurrence about one year later, a complete work-up was done, which included an ENT evaluation and a CT scan. Imaging demonstrated a predisposing anatomic variant bilaterally.	Treating physicians should know that a dehiscent facial nerve canal could be a predisposing factor to developing recurrent facial nerve baroparesis, and should conduct imaging work-ups like a CT scan.

	A dehiscent facial nerve canal may be a predisposing factor to developing recurrent facial baroparesis.	
Kerget et al. (2018) (70)	A 32-year-old female patient was diagnosed with Sarcoidosis, but with no treatment effect. In her detailed occupational history, she had a working history as an inspector in a jean sandblasting factory between 1997 and 2000. The diagnosis of silicosis was made according to the combination of occupational history and compatible radiological findings.<br>Because of male-dominance in labor, occupational history among females may be underestimated or missed.	A comprehensive occupational history should be taken in order not to miss or delay the diagnosis. Gender prejudice should not influence the occupational history-taking process.
Fiz Galende et al. (2018) (71)	A 27-year-old man was misdiagnosed as a virus infection with no treatment effect. In occupational history taking, his father referred to working history in tunnel sewers with the presence of rats in the working zones. The correct diagnosis of leptospirosis can be made thereafter.	Complete occupational history should be taken from a patient with a questionable diagnosis.
Akhter et al. (2018) (72)	Among 73 patients, Tuberculosis was treated before presentation in 28 (38.35%) of interstitial lung disease patients. Except for two silicosis patients who had smear-positive tuberculosis, rest of the patients were misdiagnosed as having tuberculosis.	General knowledge about interstitial lung diseases should be provided to primary care physicians, especially in countries with high tuberculosis burden.
Preisser et al. (2017) (73)	A technician of an incineration plant and a garbage collector (respectively) experiencing workplace-related respiratory symptoms were diagnosed as drug-resistant pneumonia. Type I and type III-sensitization to Aspergillus were present in both cases. Due to typical symptoms and chest, CT showing infiltrates and bronchiectasis, ABPA was diagnosed according to current diagnostic criteria. In addition, IgE against Penicillium species and Micropolyspora faena were identified.	Allergic bronchopulmonary aspergillosis as an occupational disease for waste management workers has not been known. Further researches for causal relationships between possible occupational or environmental exposures and health outcomes are required.
Eovaldi et al. (2015) (74)	A 23-year-old in active duty in the United States Air Force aircrew male presented to the flight medicine clinic. After experiencing a sudden onset penile pain while flying as part of a long-range operational mission. He was treated as pelvic infection, but the correct diagnosis was corpus cavernosum thrombosis.	Treating physicians should be aware of this rare condition that is associated with prolonged air travel.
Swathi et al. (2014) (75)	An intraconal foreign body was missed during the initial examination of a maxillofacial	A treating physician should know that an intraorbital foreign body

	injury and was subsequently successfully extracted by an endoscopic approach. A CT scan was essential in making a correct diagnosis.	might not be detected by typical diagnostic methods. The physician should conduct advanced imaging work-ups like a CT scan.
Laurent et al. (2014) (76)	Kappa-Weighted coefficients between trained experts ranged from 0.28 to 0.52 (fair to good), 0.59 to 0.86 (good to excellent), and 0.11 to 0.66 (poor to good) for the diagnosis of asbestosis, pleural plaques, and fibrosis of the visceral pleura, respectively.	Interpretation of benign asbestos-related thoracic abnormalities requires standardization of the reading and trained readers.
Larsen et al. (2013) (77)	A presumptive clinicopathologic diagnosis of interstitial lung disease was made in all five cases, and wedge biopsies were performed. Additional immunohistochemistry showed at least 3 positive mesothelial markers and at least 3 negative adenocarcinoma markers in all cases, consistent with mesothelioma. (Diffuse parenchymal pulmonary mesothelioma)	Recognition of this occurrence (diffuse parenchymal pulmonary mesothelioma) due to asbestos exposure is essential to avoid misdiagnosis.
Mayer et al. (2011) (78)	When the patient presented to our institution, he had DOL-approved CBD. He reported symptoms of cough and dyspnea. HRCT scan showed abnormalities more suggestive of MAI than CBD, with bronchiectasis, centrilobular nodularity, and tree-in-bud appearance in the right middle and lower lobes. Repeat bronchoscopy was pursued. The BAL cell counts revealed 43.4 million WBC and 20% lymphocytes, 72% macrophages, and 8% neutrophils with a normal BAL BeLPT. The biopsy did not reveal granulomatous inflammation. Bronchoalveolar lavage culture grew MAI.	Providing a diagnosis of chronic beryllium disease based solely on the Department of Labor criteria can cause misdiagnoses. More objective diagnostic methods should be implemented. In addition, other differential diagnoses should be considered.
Giannikas et al. (1998) (79)	The line that was initially believed to be the spiral fracture was still visible after six months of initial treatment. Computerized tomography demonstrated a sequestrum and debris within the medullary canal, suggestive of localized osteomyelitis due to a remaining piece of the grinder's blade.	Injuries caused by powerful machinery should be investigated thoroughly using a CT scan at the first instance.

Supplementary material C-1. Classification of articles according to each step of the typical framework

Each step of the typical framework	(i) Evidence of a disease	(ii) Evidence of hazardous exposures	(iii) Evidence of causal relationship
The articles classified according to each step¶	2, 4, 5, 6, 7, 8, 13, 14, 21, 25, 26, 28, 29, 31, 32, 34, 35, 41, 46 ,47, 48, 49, 51, 52, 53, 55, 56, 57, 59, 61, 64, 65, 66, 69, 72, 73, 74, 75 ,76, 77, 79	1, 3, 9, 10, 11, 12, 15, 16, 18, 19, 20, 23, 26, 30, 32, 33, 34, 38, 39, 40, 42, 43, 44, 45, 50, 54, 58, 60, 63, 67, 68, 70, 71	16, 17 , 18, 22, 24, 27, 35, 36, 37, 62, 78

¶Numbers in the third row is the reference number in this Supplementary materials.

Supplementary material C-2. Classification of articles according to each step of the causation model

Each step of the typical framework	(i) Knowledge base	(ii) Heuristics	(iii) Complete work-ups	(iv) Diagnosis	(v) Management	(vi) Feedback
The articles classified according to each step¶	9, 10, 11, 12, 14, 15, 18, 19, 20, 26, 27, 29, 30, 31, 32, 33, 35, 37, 38, 39, 40, 43, 44, 45, 47, 51, 52, 53, 54, 55, 56, 57, 58, 63, 66, 67, 69, 72, 73, 74, 75, 77	24, 36, 42, 46, 49, 50, 59, 60, 62, 64, 65, 70, 71, 78	1, 2, 3, 4, 5, 6, 7, 8, 13, 23, 28, 34, 48, 61	16, 17, 22, 41, 68, 76, 79	None	21, 25

¶Numbers in the third row is the reference number in this Supplementary materials.

Supplementary material D. The characteristics of the final included articles

Article	Study type	Study period	Population	Initial misdiagnosis	Correct diagnosis	False (+) or (-)[¶]	The specialty of diagnosing doctor (the initial misdiagnosis)	The specialty of diagnosing doctor (the correct diagnosis)	Typical framework[⁋]	Causation model[€]
Houle et al. (2012) (1)	Case series (retrospective)	2002-2011	St Michael's Hospital in Toronto, Ontario, Canada	Non-allergic irritant contact dermatitis	Allergic contact dermatitis (Occupational epoxy)	False (-)	Dermatology	Dermatology, OEM	[2] Evidence of hazardous exposures	[3] Complete work-ups
Griffin et al. (2018) (2)	Cohort study	2017-2018	A derivation and validation cohort in Guatemala	Acute kidney injury	Normal kidney function	False (+)	Nephrology, OEM	Nephrology	[1] Evidence of a disease	[3] Complete work-ups
Bruze et al. (2013) (3)	Case report	NA	Sweden	Allergic contact dermatitis (cobalt exposure)	Non-allergic contact dermatitis	False (+)	Dermatology	OEM	[2] Evidence of hazardous exposures	[3] Complete work-ups
Sastre et al. (2003) (4)	Case series (prospective)	NA	Spain	No asthmatic reaction	Occupational asthma (isocyanate)	False (-)	Allergy	Allergy	[1] Evidence of a disease	[3] Complete work-ups
Behrman et al. (2003) (5)	Case series (retrospective)	2000	Hospital workers in the same hospital in the US	Varicella-zoster virus infection	No infection	False (+)	OEM	OEM	[1] Evidence of a disease	[3] Complete work-ups
Stenton et al. (1994) (6)	Case report	NA	A nurse in a hospital in the UK	Occupational asthma	No asthmatic reaction	False (+)	OEM	OEM	[1] Evidence of a disease	[3] Complete work-ups
Inai et al. (2009) (7)	Discussion	2006-2007	Japan	Benign asbestos pleurisy	Mesothelioma	False (-)	Pathology	Pathology	[1] Evidence of a disease	[3] Complete work-ups
Ghio et al. (2014) (8)	Discussion	NA	US	Interstitial lung disease or other diseases	Interstitial lung disease or other diseases	False (+) and (-)	Pathology	Pathology	[1] Evidence of a disease	[3] Complete work-ups

Muller et al. (2006) (9)	Case-control study (prospective)	1997-2005	In a hospital in Germany	Sarcoidosis	Chronic beryllium disease	False (-)	Pulmonology and allergy	Pulmonology and allergy	[2] Evidence of hazardous exposures	[1] Knowledge base
Jacobs et al. (2005) (10)	Narrative review	NA	Published articles	Asthma or asthmatic bronchitis	Hypersensitivity pneumonitis	False (-)	NA	NA	[2] Evidence of hazardous exposures	[1] Knowledge base
Garland et al. (2004) (11)	Cohort study (prospective) A nested case-control study	1975-2001	The Navy Lung Disease Assessment Program in the US	Sarcoidosis	Occupational interstitial lung disease	False (-)	NA	Pathology	[2] Evidence of hazardous exposures	[1] Knowledge base
Fireman et al. (2003) (12)	Case series (prospective)	NA	In a hospital in Israel	Sarcoidosis	Chronic beryllium disease	False (-)	Pulmonology and allergy	Pulmonology and allergy OEM	[2] Evidence of hazardous exposures	[1] Knowledge base
Kucenic et al. (2002) (13)	Case series (retrospective)	1994-1999	In a hospital in the US	Non-allergic irritant contact dermatitis	Allergic contact dermatitis (Occupational allergen)	False (-)	Dermatology OEM	Dermatology OEM	[1] Evidence of a disease	[3] Complete work-ups
Hartman et al. (1998) (14)	Narrative review	NA	Published articles	Neurotoxicant exposure or primary psychiatric illness and multiple chemical sensitivity	Neurotoxicant exposure or primary psychiatric illness and multiple chemical sensitivity	False (+) and (-)	NA	NA	[1] Evidence of a disease	[1] Knowledge base
Kotloff et al. (1993) (15)	Case report	1989	A dental laboratory technician in the US	Sarcoidosis	Chronic beryllium disease	False (-)	Pulmonology and critical care	Pulmonology and critical care	[2] Evidence of hazardous exposures	[1] Knowledge base
Igata et al. (1993) (16)	Case series (prospective)	1990	Minamata city in Japan	Minamata disease or other diseases	Minamata disease or other diseases	False (+) and (-)	OEM	OEM	[3] Evidence of causal relationship	[4] Diagnosis
Black et al. (1993) (17)	Case series (prospective)	NA	US	Environmental illnesses or other diseases	Environmental illnesses or other diseases	False (+) and (-)	Psychiatry	Psychiatry	[3] Evidence of causal relationship	[4] Diagnosis
Kuratsune et al.	Case-control	1967-	Japan	Typical lung cancer	Occupational lung	False (-)	Pulmonology	Public health	[2]	[1]

(1974) (18)	study	1969			cancer				Evidence of hazardous exposures	Knowledge base
Kerget et al. (2019) (19)	Case report	2012	Turkey	Other respiratory diseases	Silicosis	False (-)	Pulmonology	Pulmonology Radiology Pathology	[2] Evidence of hazardous exposures	[1] Knowledge base
He et al. (2019) (20)	Case report	NA	China	Other febrile diseases.	Tsutsugamushi disease (occupational origin)	False (-)	NA	NA	[2] Evidence of hazardous exposures	[1] Knowledge base
Wuellner et al. (2018) (21)	Interview investigation	2013-2014	US	Occupational injury and illness or Other diseases of non-occupational origin	Occupational injury and illness or Other diseases of non-occupational origin	False (+) and (-)	NA	NA	[1] Evidence of a disease	[6] Feedback
Waljee et al. (2018) (22)	Discussion	NA	US	Carpal tunnel syndrome	Nonspecific activity-related arm pain	False (+)	Orthopedics	Orthopedics	[3] Evidence of causal relationship	[4] Diagnosis
Dupas et al. (2013) (23)	Discussion	2007-2010	France	Asthma or allergic condition	Multiple chemical sensitivity	False (-)	NA	NA	[2] Evidence of hazardous exposures	[3] Complete work-ups
Schaumburg et al. (2005) (24)	Case series	NA	US	Neurotoxic disease	A naturally occurring nervous system disease, psychogenic illness	False (+)	Neurology	Neurology	[3] Evidence of causal relationship	[2] Heuristics
Egilman et al. (2004) (25)	Case report	1998	US	Asbestos-related malignant cancer	Bilateral parietal pleural plaque	False (+)	NA	NA	[1] Evidence of a disease	[6] Feedback
Menezes et al. (2003) (26)	Case report	NA	India	Other diseases	Lead poisoning (battery worker)	False (-)	Biochemistry	Biochemistry	[2] Evidence of hazardous exposures	[1] Knowledge base
Morgan et al. (1999) (27)	Discussion	NA	UK	Other diseases	Occupational diseases, general	False (-)	NA	NA	[3] Evidence of causal	[1] Knowledge base

									relationship	
Corbett et al. (1999) (28)	Case series (retrospective)	1996-1997	South Africa	Pneumoconiosis or other lung diseases	Pneumoconiosis or other lung diseases	False (+) and (-)	Radiology	Pathology	[1] Evidence of a disease	[3] Complete work-ups
Hinchcliffe et al. (1997) (29)	Narrative review	NA	Published articles	Occupational noise-induced hearing loss or other hearing loss disease	Occupational noise-induced hearing loss or other hearing loss disease	False (+) and (-)	NA	NA	[1] Evidence of a disease	[1] Knowledge base
Laczniak et al. (2014) (30)	Exposure assessment	NA	US	Sarcoidosis	Chronic beryllium disease	False (-)	NA	NA	[2] Evidence of hazardous exposures	[1] Knowledge base
Riario Sforza et al. (2017) (31)	Narrative review	NA	Published articles	Idiopathic interstitial lung disease	Hypersensitivity pneumonitis	False (-)	NA	NA	[1] Evidence of a disease	[1] Knowledge base
Kadu et al. (2012) (32)	Narrative review	NA	Published articles	Other diseases	Lead poisoning	False (-)	NA	NA	[2] Evidence of hazardous exposures	[1] Knowledge base
Glazer et al. (2011) (33)	Narrative review	NA	Published articles	Idiopathic pulmonary fibrosis	Occupational or environmental interstitial lung disease	False (-)	NA	NA	[2] Evidence of hazardous exposures	[1] Knowledge base
Ansley et al. (2012) (34)	Case series (prospective)	2009-2010	UK	Asthma or exercise-induced bronchoconstriction	No lung function abnormality	False (+)	Sport and exercise science	Sport and exercise science	[1] Evidence of a disease	[3] Complete work-ups
Dudarev et al. (2013) (35)	Database analysis	1980-2010	Norway	Other diseases	Occupational diseases, general	False (-)	NA	NA	[3] Evidence of causal relationship	[1] Knowledge base
Kahan et al. (1996) (36)	Interview investigation	NA	Israel	Other diseases	Occupational diseases, general	False (-)	NA	NA	[3] Evidence of causal relationship	[2] Heuristics
Constantin et al. (2015) (37)	Case series (retrospective)	2000-2004	Romania	Other respiratory diseases	Occupational asthma	False (-)	NA	NA	[3] Evidence of causal	[1] Knowledge base

									relationship	
Guarnieri et al. (2019) (38)	Case series	2016-2017	Italy	Sarcoidosis	Silicosis	False (-)	Cardiology	OEM	[2] Evidence of hazardous exposures	[1] Knowledge base
Ross et al. (2016) (39)	Case series	2012	UK	Other diseases	Methyl iodide and manganese poisoning	False (-)	Emergency	Psychiatry	[2] Evidence of hazardous exposures	[1] Knowledge base
Zhang et al. (2014) (40)	Case series	NA	China	Other diseases	N-hexane poisoning	False (-)	NA	NA	[2] Evidence of hazardous exposures	[1] Knowledge base
Lu et al. (2013) (41)	Narrative review	1985-2013	Published articles	Other lung diseases	Pneumoconiosis or silicotuberculosis	False (-)	NA	NA	[1] Evidence of a disease	[4] Diagnosis
Sauler et al. (2012) (42)	Discussion	NA	US	Other lung diseases	Chronic terminal airways and parenchymal lung disease	False (-)	NA	NA	[2] Evidence of hazardous exposures	[2] Heuristics
Baur et al. (2016) (43)	Case report	NA	Germany	Sarcoidosis	Chronic beryllium disease	False (-)	NA	NA	[2] Evidence of hazardous exposures	[1] Knowledge base
Rodriguez et al. (2005) (44)	Case report	NA	Mexico	Motor dysfunction disease	Musician’s focal dystonia	False (-)	Rheumatology	Rheumatology	[2] Evidence of hazardous exposures	[1] Knowledge base
Shusterman et al. (1993) (45)	Narrative review	NA	US	Viral flu	Polymer fume fever	False (-)	NA	NA	[2] Evidence of hazardous exposures	[1] Knowledge base
Ryan et al. (1993) (46)	Case report	NA	US	Ulnar styloid fracture	Calcific tendinitis of the flexor carpi ulnaris	False (-)	Orthopedics	Orthopedics	[1] Evidence of a disease	[2] Heuristics
Tezer et al. (2011) (47)	Case series	NA	Turkey	A contagious infectious disease	Mercury poisoning	False (-)	Pediatrics	Pediatrics	[1] Evidence of	[1] Knowledge

									a disease	base
Tonini et al. (2009) (48)	Case report	NA	Italy	Asthma	Irritant vocal cord dysfunction	False (-)	Pulmonology	OEM	[1] Evidence of a disease	[3] Complete work-ups
Chirico et al. (2016) (49)	National statistics analysis	1996-2011	Italy	Other psychiatric or stress disorder	Workplace adjustment disorder	False (-)	NA	NA	[1] Evidence of a disease	[2] Heuristics
Kales et al. (2006) (50)	Case series	2000-2003	US	Allergic reactions to natural rubber latex	Other diseases	False (+)	NA	NA	[2] Evidence of hazardous exposures	[2] Heuristics
Randhawa et al. (2010) (51)	Case series	NA	UK	Laryngopharyngeal reflux	Allergic laryngitis (Occupational origin)	False (-)	Otorhinolaryngolo gy	Otorhinolaryngolo gy	[1] Evidence of a disease	[1] Knowledge base
Ohtani et al. (2008) (52)	Case series	2001-2002	Japan	Idiopathic interstitial pneumonia	Chronic summer-type hypersensitivity pneumonitis	False (-)	Pulmonology	Internal medicine	[1] Evidence of a disease	[1] Knowledge base
Chan et al. (2008) (53)	Case report	NA	Ireland	Occupational overuse syndrome	Guyon's canal syndrome	False (+)	General practitioner	Hand surgery	[1] Evidence of a disease	[1] Knowledge base
Taskar et al. (2006) (54)	Narrative review	1990-2006	Published articles	Idiopathic pulmonary fibrosis	Interstitial lung disease (Occupational or Environmental origin)	False (+) and (-)	NA	NA	[2] Evidence of hazardous exposures	[1] Knowledge base
Liu et al. (2011) (55)	Case series	2000-2010	China	Other diseases	Mercury poisoning	False (-)	NA	NA	[1] Evidence of a disease	[1] Knowledge base
Galdi et al. (2005) (56)	Case report	2002	Italy	Reactive airway dysfunction syndrome	Irritant vocal cord dysfunction	False (-)	Pulmonology	Otorhinolaryngolo gy OEM	[1] Evidence of a disease	[1] Knowledge base
Mirzaei et al. (2019) (57)	Case report	NA	Iran	Fractured orbital bone	A plastic foreign body (Occupational craniofacial injury)	False (-)	Neurosurgery	Neurosurgery	[1] Evidence of a disease	[1] Knowledge base
Papakonstantinou et al. (2017) (58)	Case report	NA	UK	Toxoplasmosis	Whipple’s disease	False (-)	Infection	Pathology	[2] Evidence of hazardous	[1] Knowledge base

									exposures	
Mouawad et al. (2014) (59)	Case report	NA	US	Thoracic aortic rupture or dissection	Malignant mesothelioma	False (-)	Emergency department Vascular surgery	Vascular surgery	[1] Evidence of a disease	[2] Heuristics
Poole et al. (2008) (60)	Case report	NA	UK	Hand-arm vibration syndrome	Cold hemagglutinin disease	False (+)	OEM	OEM Hematology	[2] Evidence of hazardous exposures	[2] Heuristics
Walusiak et al. (2002) (61)	Case report	NA	Poland	Bronchial asthma (occupational origin)	Carcinoid syndrome	False (+)	Pulmonology	OEM	[1] Evidence of a disease	[3] Complete work-ups
Greve et al. (2006) (62)	Case series	NA	US	Toxic exposure	Cognitive malingering	False (+)	OEM	Psychiatry	[3] Evidence of causal relationship	[2] Heuristics
Weber et al. (1999) (63)	Case report	NA	US	An insect bite or Child abuse	Phytophotodermatitis	False (-)	Emergency department	Emergency department	[2] Evidence of hazardous exposures	[1] Knowledge base
Rosal-Sanchez et al. (2002) (64)	Case report	NA	Spain	Tuberculosis	Pigeon fancier’s lung	False (-)	NA	NA	[1] Evidence of a disease	[2] Heuristics
Greenspan et al. (1991) (65)	Narrative review	NA	Published articles	Other bone abnormalities	Condensing osteitis of the clavicle	False (-)	NA	NA	[1] Evidence of a disease	[2] Heuristics
Mazzei et al. (2019) (66)	Case series	2009-2017	Italy	Other lung diseases	Occupational interstitial lung diseases	False (-)	Radiology OEM	Radiology OEM	[1] Evidence of a disease	[1] Knowledge base
Center for Disease Control and Prevention (2013) (67)	Case report	2007-2009	US	Other lung diseases	Obliterative bronchiolitis (Occupational)	False (-)	Pulmonology OEM	Pulmonology OEM	[2] Evidence of hazardous exposures	[1] Knowledge base
Bouzgarrou et al. (2020) (68)	Case series	NA (6 years)	Tunisia	Allergic contact dermatitis	Occupational irritant contact dermatitis	False (-)	NA	OEM	[2] Evidence of hazardous exposures	[4] Diagnosis
Marinides et al.	Case report	NA	US	Arterial gas embolism	Facial nerve baroparesis	False (-)	NA	OEM	[1]	[1]

(2019) (69)									Evidence of a disease	Knowledge base
Kerget et al. (2018) (70)	Case report	NA	Turkey	Sarcoidosis	Silicosis	False (-)	Pulmonology	Pulmonology	[2] Evidence of hazardous exposures	[2] Heuristics
Fiz Galende et al. (2018) (71)	Case series	NA	Spain	Virus infection	Leptospirosis	False (-)	NA	NA	[2] Evidence of hazardous exposures	[2] Heuristics
Akhter et al. (2018) (72)	Case series (prospective)	2017	Pakistan	Tuberculosis	Interstitial lung disease	False (-)	Pulmonology	Pulmonology	[1] Evidence of a disease	[1] Knowledge base
Preisser et al. (2017) (73)	Cohort study (prospective)	2012	Germany	Drug-resistant pneumonia	Allergic bronchopulmonary aspergillosis	False (-)	NA	OEM	[1] Evidence of a disease	[1] Knowledge base
Eovaldi et al. (2015) (74)	Case report	NA	US	Pelvic infection	Corpus cavernosum thrombosis	False (-)	Emergency department	Emergency department	[1] Evidence of a disease	[1] Knowledge base
Swathi et al. (2014) (75)	Case report	NA	India	Retrobulbar hemorrhage/edema	Intraconal foreign body	False (-)	Opthalmology	Opthalmology	[1] Evidence of a disease	[1] Knowledge base
Laurent et al. (2014) (76)	Cohort study (prospective)	2003-2005	France	Asbestosis<br>Pleural plaques<br>Fibrosis of the visceral pleura	Asbestosis<br>Pleural plaques<br>Fibrosis of the visceral pleura	False (+) and (-)	Pulmonology<br>OEM	Pulmonology<br>OEM	[1] Evidence of a disease	[4] Diagnosis
Larsen et al. (2013) (77)	Case series	2010-2012	US	Interstitial lung disease	Diffuse parenchymal pulmonary mesothelioma	False (-)	NA	Pathology	[1] Evidence of a disease	[1] Knowledge base
Mayer et al. (2011) (78)	Case report	NA	US	Chronic beryllium disease	Mycobacterium Avium-Intracellulare infection	False (+)	NA	NA	[3] Evidence of causal relationship	[2] Heuristics
Giannikas et al. (1998) (79)	Case report	NA	UK	A spiral fracture of the right tibia	Unrecognized foreign body (a piece of the grinder's blade)	False (-)	Orthopedics	Orthopedics	[1] Evidence of a disease	[4] Diagnosis

NA: Not Available in the article text
¶False (+) or (-) for Occupational Disease or Environmental Disease Category
₱Typical framework in the Methods section 2.5. Typical framework for the diagnosis of OD and ED
€Causation model in the Methods section 2.6. Causation model for misdiagnosis of OD or ED

REFERENCES

1. Houle M-C, Holness LD, DeKoven J, Skotnicki S. Additive value of patch testing custom epoxy materials from the workplace at the Occupational Disease Specialty Clinic in Toronto. Dermatitis. 2012;23(5):214-9.

2. Griffin BR, Butler-Dawson J, Dally M, Krisher L, Cruz A, Weitzenkamp D, et al. Unadjusted point of care creatinine results overestimate acute kidney injury incidence during field testing in Guatemala. PloS one. 2018;13(9):e0204614.

3. Bruze M, Hamada H, Dahlin J, Duner K, Persson L. A positive cobalt spot test falsely indicating an occupational allergic contact dermatitis caused by cobalt. Contact dermatitis. 2013;69(3):172-5.

4. Sastre J, Fernandez-Nieto M, Novalbos A, De Las Heras M, Cuesta J, Quirce S. Need for monitoring nonspecific bronchial hyperresponsiveness before and after isocyanate inhalation challenge. Chest. 2003;123(4):1276-9.

5. Behrman A, Schmid DS, Crivaro A, Watson B. A cluster of primary varicella cases among healthcare workers with false-positive varicella zoster virus titers. Infect Control Hosp Epidemiol. 2003;24(3):202-6.

6. Stenton SC, Beach JR, Dennis JH, Keaney NP, Hendrick DJ. Glutaraldehyde, asthma and work--a cautionary tale. Occupational medicine (Oxford, England). 1994;44(2):95-8.

7. Inai K. Diagnosis of Asbestos exposure-related diseases based on pathological features. Japanese Journal of Lung Cancer. 2009;49(1):83-7.

8. Ghio A, Sangani R, Roggli V. Expanding the spectrum of particle-and fiber-associated interstitial lung diseases. Turk Toraks Dergisi. 2014;15(1):1-8.

9. Müller-Quernheim J, Gaede K, Fireman E, Zissel G. Diagnoses of chronic beryllium disease within cohorts of sarcoidosis patients. European Respiratory Journal. 2006;27(6):1190-5.

10. Jacobs RL, Andrews CP, Coalson JJ. Hypersensitivity pneumonitis: beyond classic occupational disease–changing concepts of diagnosis and management. Annals of Allergy, Asthma &

Immunology. 2005;95(2):115-28.

11. Garland FC, Gorham ED, Kaiser K, Travis WD, Centeno JA, Abraham JL, et al. Navy Lung Disease Assessment Program. NAVAL HEALTH RESEARCH CENTER SAN DIEGO CA; 2004.

12. Fireman E, Haimsky E, Noiderfer M, Priel I, Lerman Y. Misdiagnosis of sarcoidosis in patients with chronic beryllium disease. Sarcoidosis, vasculitis, and diffuse lung diseases: official journal of WASOG. 2003;20(2):144-8.

13. Kucenic MJ, Belsito DV. Occupational allergic contact dermatitis is more prevalent than irritant contact dermatitis: a 5-year study. Journal of the American Academy of Dermatology. 2002;46(5):695-9.

14. Hartman DE. Missed diagnoses and misdiagnoses of environmental toxicant exposure: the psychiatry of toxic exposure and multiple chemical sensitivity. Psychiatric Clinics of North America. 1998;21(3):659-70.

15. Kotloff RM, Richman PS, Greenacre JK, Rossman MD. Chronic beryllium disease in a dental laboratory technician. American Review of Respiratory Disease. 1993;147(1):205-7.

16. Igata A. Epidemiologic and clinical features of Minamata disease. Environmental Research. 1993;63(1):157-69.

17. Black DW. Environmental illness and misdiagnosis-a growing problem. Regulatory Toxicology and Pharmacology. 1993;18(1):23-31.

18. Kuratsune M, Tokudome S, Shirakusa T, Yoshida M, Tokumitsu Y, Hayano T, et al. Occupational lung cancer among copper smelters. International journal of cancer. 1974;13(4):552-8.

19. Kerget B, Araz O, Yilmazel Ucar E, Karaman A, Calik M, Alper F, et al. Female workers' silicosis diagnosis delayed due to gender bias. Occupational medicine (Oxford, England). 2019;69(3):219-22.

20. He WJ, Wang DQ, Zhang PP, Fu PL, Li ZJ. [The importance of occupational history in clinical thinking from the diagnosis and treatment of a case of Tsutsugamushi disease]. Zhonghua lao dong wei sheng zhi ye bing za zhi = Zhonghua laodong weisheng zhiyebing zazhi = Chinese journal of industrial hygiene and occupational diseases. 2019;37(8):639-41.

21. Wuellner S, Phipps P. Employer knowledge of federal requirements for recording work-related injuries and illnesses: Implications for occupational injury surveillance data. American journal of industrial medicine. 2018;61(5):422-35.

22. Waljee JF, Ring D. Diagnosis and Treatment of Carpal Tunnel Syndrome in Low-prevalence Circumstances. The Journal of the American Academy of Orthopaedic Surgeons. 2018;26(16):573-5.

23. Dupas D, Dagorne MA. [Multiple chemical sensitivity: a diagnosis not to be missed]. Revue des maladies respiratoires. 2013;30(2):99-104.

24. Schaumburg HH, Albers JW. Pseudoneurotoxic disease. Neurology. 2005;65(1):22-6.

25. Egilman D, Rankin Bohme S. Attorney-directed screenings can be hazardous. American journal of industrial medicine. 2004;45(3):305-7.

26. Menezes G, D'Souza H S, Venkatesh T. Chronic lead poisoning in an adult battery worker. Occupational medicine (Oxford, England). 2003;53(7):476-8.

27. Morgan DR. The general practitioners' view. Occupational medicine (Oxford, England). 1999;49(6):403-5.

28. Corbett EL, Murray J, Churchyard GJ, Herselman PC, Clayton TC, De Cock KM, et al. Use of miniradiographs to detect silicosis. Comparison of radiological with autopsy findings. Am J Respir Crit Care Med. 1999;160(6):2012-7.

29. Hinchcliffe R. Medical examiner variability. J Laryngol Otol. 1997;111(1):8-14.

30. Laczniak AN, Gross NA, Fuortes LJ, Field RW. Unsuspected exposure to beryllium: potential implications for sarcoidosis diagnoses. Sarcoidosis Vasc Diffuse Lung Dis. 2014;31(2):163-9.

31. Riario Sforza GG, Marinou A. Hypersensitivity pneumonitis: A complex lung disease. Clinical and Molecular Allergy. 2017;15(1).

32. Kadu AS, Nampalliwar AR, Pandey AG, Sharma A, Gothecha VK. Lead poisoning: An overlooked diagnosis in clinical practice. International Journal of Research in Ayurveda and Pharmacy. 2012;3(5):639-44.

33. Glazer CS. Occupation, avocation, and interstitial lung disease. Clinical Pulmonary Medicine. 2011;18(1):20-8.

34. Ansley L, Kippelen P, Dickinson J, Hull J. Misdiagnosis of exercise-induced bronchoconstriction in professional soccer players. Allergy. 2012;67(3):390-5.

35. Dudarev AA, Talykova LV, Odland JO. Occupational diseases in Murmansk Oblast: 1980-2010. International journal of circumpolar health. 2013;72:20468.

36. Kahan E, Weingarten MA, Appelbaum T. Attitudes of primary care physicians to the management of asthma and their perception of its relationship to patients' work. Isr J Med Sci. 1996;32(9):757-62.

37. Constantin B, Postolache P, Croitoru A, Nemes R. Occupational Bronchial Asthma-Clinical and Epidemiological Aspects. Journal of Environmental Protection and Ecology. 2015;16(2):517-20.

38. Guarnieri G, Bizzotto R, Gottardo O, Velo E, Cassaro M, Vio S, et al. Multiorgan accelerated silicosis misdiagnosed as sarcoidosis in two workers exposed to quartz conglomerate dust. Occup Environ Med. 2019;76(3):178-80.

39. Ross SM. Delayed cognitive and psychiatric symptoms following methyl iodide and manganese poisoning: Potential for misdiagnosis. Cortex. 2016;74:427-39.

40. Zhang J, Li Z, Wang J, Li H, Si T, Deng L, et al. Misdiagnosis of occupational chronic n-hexane poisoning: an analysis of 16 cases. Zhonghua lao dong wei sheng zhi ye bing za zhi= Zhonghua laodong weisheng zhiyebing zazhi= Chinese journal of industrial hygiene and occupational diseases. 2014;32(12):930-1.

41. Lü X, Wang H. Misdiagnosis of pneumoconiosis or silicotuberculosis in China: a pooled analysis of 1178 cases. Zhonghua lao dong wei sheng zhi ye bing za zhi= Zhonghua laodong weisheng zhiyebing zazhi= Chinese journal of industrial hygiene and occupational diseases. 2013;31(8):564-7.

42. Sauler M, Gulati M. Newly recognized occupational and environmental causes of chronic terminal airways and parenchymal lung disease. Clinics in chest medicine. 2012;33(4):667-80.

43. Baur X, Muller-Quernheim J. [Case Report with Differential Diagnostic Aspects of Sarcoidosis]. Pneumologie (Stuttgart, Germany). 2016;70(3):201-4.

44. Rodriguez AV, Kooh M, Guerra LA, Martinez-Lavin M, Pineda C. Musician's cramp: a case

report and literature review. Journal of clinical rheumatology : practical reports on rheumatic & musculoskeletal diseases. 2005;11(5):274-6.

45. Shusterman DJ. Polymer fume fever and other fluorocarbon pyrolysis-related syndromes. Occup Med. 1993;8(3):519-31.

46. Ryan WG. Calcific tendinitis of flexor carpi ulnaris: an easy misdiagnosis. Arch Emerg Med. 1993;10(4):321-3.

47. Tezer H, Erkocoglu M, Kara A, Bayrakci B, Duzova A, Teksam O, et al. Household poisoning cases from mercury brought from school. European journal of pediatrics. 2011;170(3):397-400.

48. Tonini S, Dellabianca A, Costa C, Lanfranco A, Scafa F, Candura SM. Irritant vocal cord dysfunction and occupational bronchial asthma: differential diagnosis in a health care worker. International journal of occupational medicine and environmental health. 2009;22(4):401-6.

49. Chirico F. Adjustment disorder as an occupational disease: Our experience in Italy. Int J Occup Environ Med (The IJOEM). 2016;7(1 January):716-52-7.

50. Kales SN, Lee EC. Pseudo-latex allergy associated with "latex" paint exposure: a potential cause of iatrogenic disability. Journal of occupational and environmental medicine. 2006;48(1):83-8.

51. Randhawa PS, Mansuri S, Rubin JS. Is dysphonia due to allergic laryngitis being misdiagnosed as laryngopharyngeal reflux? Logopedics Phoniatrics Vocology. 2010;35(1):1-5.

52. Ohtani Y, Ochi J, Mitaka K, Takemura T, Jinta T, Kuramochi J, et al. Chronic summer-type hypersensitivity pneumonitis initially misdiagnosed as idiopathic interstitial pneumonia. Internal Medicine. 2008;47(9):857-62.

53. Chan JC, Tiong WH, Hennessy MJ, Kelly JL. A Guyon's canal ganglion presenting as occupational overuse syndrome: A case report. Journal of brachial plexus and peripheral nerve injury. 2008;3(1):4.

54. Taskar VS, Coultas DB. Is idiopathic pulmonary fibrosis an environmental disease? Proceedings of the American Thoracic Society. 2006;3(4):293-8.

55. Liu XL, Wang HB, Sun CW, Xiong XS, Chen Z, Li ZS, et al. [The clinical analysis of

mercury poisoning in 92 cases]. Zhonghua nei ke za zhi. 2011;50(8):687-9.

56. Galdi E, Perfetti L, Pagella F, Bertino G, Ferrari M, Moscato G. Irritant vocal cord dysfunction at first misdiagnosed as reactive airway dysfunction syndrome. Scand J Work Environ Health. 2005;31(3):224-6.

57. Mirzaei F, Salehpour F, Shokuhi G, Asvadi Kermani T, Salehi S, Parsay S. An unusual case of intra orbital foreign body; diagnosis, management, and outcome: a case report. BMC surgery. 2019;19(1):76.

58. Papakonstantinou D, Riste MJ, Langman G, Moran E. Misdiagnosing Whipple's disease in the young. BMJ case reports. 2017;2017.

59. Mouawad NJ, Daniel VC, Starr JE. Advanced malignant mesothelioma mimicking acute contained thoracic aortic rupture. Interactive cardiovascular and thoracic surgery. 2014;18(2):242-4.

60. Poole CJ. Cold haemagglutinin disease misdiagnosed as hand-arm vibration syndrome. Occupational medicine (Oxford, England). 2008;58(3):219-21.

61. Walusiak J, Palczynski C. Carcinoid behind baker's asthma. Allergy. 2002;57(10):966-7.

62. Greve KW, Bianchini KJ, Black FW, Heinly MT, Love JM, Swift DA, et al. The prevalence of cognitive malingering in persons reporting exposure to occupational and environmental substances. Neurotoxicology. 2006;27(6):940-50.

63. Weber IC, Davis CP, Greeson DM. Phytophotodermatitis: the other “lime” disease. The Journal of emergency medicine. 1999;17(2):235-7.

64. Rosal-Sanchez M, Alvarez J, Torres MJ, Mayorga C, Perez J, Blanca M. Pigeon Fancier's Lung after low exposure. Allergy. 2002;57(7):649.

65. Greenspan A, Gerscovich E, Szabo RM, Matthews 2nd J. Condensing osteitis of the clavicle: a rare but frequently misdiagnosed condition. AJR American journal of roentgenology. 1991;156(5):1011-5.

66. Mazzei MA, Sartorelli P, Bagnacci G, Gentili F, Sisinni AG, Fausto A, et al. Occupational Lung Diseases: Underreported Diagnosis in Radiological Practice. Seminars in ultrasound, CT, and MR. 2019;40(1):36-50.

67. Centers for Disease C, Prevention. Obliterative bronchiolitis in workers in a coffee-processing facility - Texas, 2008-2012. MMWR Morbidity and mortality weekly report. 2013;62(16):305-7.

68. Bouzgarrou L, Omrane A, Chebbeh W, Boussarssar I, Kellala I, Belkhiria K, et al. Diagnosis mistaken: a non-negligible reason for rejecting the declaration of occupational dermatitis. World Allergy Organization Journal. 2020;13(8).

69. Marinides Z, Virgilio GVR. Recurrent facial nerve baroparesis in a military diver: a case report. Undersea Hyperb Med. 2019;46(1):87-90.

70. Kerget B, Araz O, Ucar EY, Karaman A, Alper F, Akgun M. Delayed Diagnosis of Silicosis to Due Denim Sandblasting in a Female Worker. American journal of respiratory and critical care medicine. 2018;197(MeetingAbstracts).

71. Fiz Galende J, Dominguez Rodriguez Y, Candel Gonzalez FJ, Cuervo Pinto R. Report of a case of leptospirosis: the importance of asking the patient about workplace factors. Emergencias : revista de la Sociedad Espanola de Medicina de Emergencias. 2018;30(1):63-4.

72. Akhter N, Rizvi NA. Interstitial Lung Diseases Misdiagnosed as Tuberculosis. Pak J Med Sci. 2018;34(2):338-41.

73. Preisser AM, Harth V. Allergic bronchopulmonary aspergillosis as an occupational disease - special risk in waste management. European Respiratory Journal. 2017;50.

74. Eovaldi B, Dunn DP. Case report: corpus cavernosum thrombosis occurring during a long-range aviation mission initially diagnosed as lymphoma. Military medicine. 2015;180(5):e608-10.

75. Swathi N, Umadevi J. An undetected intraorbital foreign body after a "trivial" facial injury. The Journal of craniofacial surgery. 2014;25(5):1782-3.

76. Laurent F, Paris C, Ferretti GR, Beigelman C, Montaudon M, Latrabe V, et al. Inter-reader agreement in HRCT detection of pleural plaques and asbestosis in participants with previous occupational exposure to asbestos. Occupational and environmental medicine. 2014;71(12):865-70.

77. Larsen BT, Klein JRH, Hornychova H, Nuti R, Thirumala S, Leslie KO, et al. Pulmonary. Laboratory Investigation. 2013;93(S1):449-71.

78. Mayer A, Maier L, Gottschall EB. Mycobacterium Avium-Intracellulare Infection And Department Of Labor Criteria For Chronic Beryllium Disease. American journal of respiratory and critical care medicine. 2011;183(1).

79. Giannikas KA, Livesley PJ. Penetrating puncture wound due to a grinding machine: a challenge in diagnosis. The Journal of trauma. 1998;44(2):404-5.
